# POU2F2‐IL‐31 Autoregulatory Circuit Converts Hepatocytes into the Origin Cells of Hepatocellular Carcinoma

**DOI:** 10.1002/advs.202004683

**Published:** 2021-05-02

**Authors:** Chunwang Yuan, Lijun Pang, Wenjing Wang, Yabo Ouyang, Xianghua Guo, Kai Liu

**Affiliations:** ^1^ Capital Medical University Affiliated to Beijing You An Hospital Beijing 100069 China; ^2^ Beijing Institute of Hepatology Beijing 100069 China

**Keywords:** hepatocellular carcinoma, IL‐31, liver cancer stem cells, origin cells, p53, POU2F2

## Abstract

Hepatocellular carcinoma (HCC) originates from fully differentiated hepatocytes, but the decisive events for converting hepatocytes to the cells of origin for HCC are still unclear. Liver cancer stem cells (LCSCs) cause HCC but are not bona fide cells of origin. Here, the expressions of POU2F2 and IL‐31 are identified in macroscopically normal livers of diethylnitrosamine‐challenged mice. An autoregulatory circuit formed by mutual induction between POU2F2 and IL‐31 drives hepatocytes to progress to LCSCs by acquiring stemness, as well as stimulates them to in vivo grow and malignantly progress. The development of the autoregulatory circuit is a decisive event for converting hepatocytes into the cells of origin, since hepatocytes expressing the circuit have acquired tumorigenic potential before progressing to LCSCs. Nonetheless, acquiring stemness is still required for the cells of origin to initiate hepatocarcinogenesis. The circuit also occurs in human cirrhotic tissues, partially elucidating how premalignant lesions progress to HCC.

## Graphical abstract

POU2F2 and IL‐31 form an autoregulatory circuit that functionally promotes fully differentiated hepatocytes to be converted into liver cancer stem cells by inducing pluripotent transcription factors such as NANOG, OCT4, and SOX2, as well as suppressing p53.

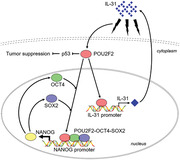

## 1 Introduction

Liver cancer is the sixth most commonly diagnosed cancer and the second most frequent cause of cancer death in men worldwide.^[^ ^1^ ^]^ Hepatocellular carcinoma (HCC) represents the major histological subtype, accounting for ≈75–85% of primary liver cancer cases.^[^ ^1^ ^]^ Most HCC cases are diagnosed at an advanced stage due to the lack of diagnostic markers and risk assessment in the clinic, contributing to the loss of a surgical resection opportunity. And even worse, chemotherapy and radiotherapy have not significant therapeutic effect on most advanced HCC cases. HCC originates from fully differentiated pericentral hepatocytes.^[^ ^2^ ^]^ Although the most prominent factors contributing to liver damage, such as hepatitis B and C viral infection, chronic alcohol consumption, and aflatoxin‐B1‐contaminated food, are well‐established inducers of HCC,^[^ ^3^ ^]^ how damaged differentiated hepatocytes are transformed to serve as the cells of origin for HCC is still unclear. Cells of origin are defined as the normal cells that acquire the first genetic hit or hits which culminate in the initiation of cancer. Functionally, they are bona fide cancer‐initiating cells that form future clinical tumors.^[^ ^4^, ^5^ ^]^ Defining the cell of origin can be critical for understanding both the environments that are permissive for transformation and the signals required for transformation.^[^ ^6^ ^]^

Cells of origin can acquire stemness and then progress to cancer stem cells (CSCs), which are defined by two attributes including self‐renewal and multipotency, during neoplastic progression.^[^ ^4^, ^6^ ^]^ CSCs contribute to carcinogenesis due to their tumorigenic capacity. The expression of pluripotent transcriptional factors (TFs) is necessary for inducing and maintaining cell stemness.^[^ ^6^ ^]^ As three core pluripotent TFs, OCT4, SOX2, and NANOG induce each other and are often used as CSC markers in many tumor types, including HCC.^[^ ^6^ ^]^ Their high expression is closely related to poor prognosis of HCC.^[^ ^7^ ^]^ p53 plays a crucial role in preventing differentiated hepatocytes from malignant transformation by mediating genome stability surveillance, cell‐cycle arrest, apoptosis, and stemness suppression.^[^ ^8^, ^9^ ^]^ When damaged hepatocytes override p53‐mediated tumor suppression, they are converted to HCC progenitor cells (HcPCs), which have acquired stemness and tumorigenic potential.^[^ ^8^, ^9^ ^]^ p53 also suppresses liver CSCs (LCSCs) development by suppressing OCT4‐SOX2 complex‐transactivated NANOG expression.^[^ ^10^ ^]^ Nonetheless, the decisive events that initiate hepatocytes to be converted into cells of origin and then to acquire stemness are hitherto unclear.

POU class 2 homeobox 2 (POU2F2) has been regarded as a B‐cell‐specific octamer TF that regulates B cell proliferation and differentiation.^[^ ^11^, ^12^, ^13^, ^14^ ^]^ It was recently shown to promote solid tumors' progression and metastasis, including pancreatic cancer, gastric cancer, and breast cancer.^[^ ^15^, ^16^, ^17^, ^18^ ^]^ We once determined POU2F2 expression in primary human hepatoma cells but not in primary normal human hepatocytes by RNA‐sequencing assay (data not shown), suggesting a link between POU2F2 expression and HCC. Here, POU2F2 expression was identified in macroscopically normal livers of mice challenged by diethylnitrosamine (DEN). As a hepatic procarcinogen, DEN induces poorly differentiated HCC nodules in mice by corrupting genome stability.^[^ ^19^ ^]^ POU2F2 transactivated NANOG expression by suppressing p53 and then induced more CSCs‐related genes expression, making POU2F2^+^ hepatocytes progress to LCSCs. Therefore, POU2F2 expression may be the decisive event that initiates hepatocytes to acquire stemness. As a type of immunodeficient mice, NOD/Shi‐scid/Il2r–/– (NOG) mouse is commonly used for analyzing in vivo tumorigenic abilities of tumor cells.^[^ ^20^ ^]^ Surprisingly, POU2F2^+^ hepatocytes could form xenograft tumors in NOG mice before they acquired stemness, demonstrating that POU2F2^+^ hepatocytes may be the cells of origin for HCC.

IL‐31, as a member of the IL‐6‐type family, is mainly produced by immune cells, epidermal keratinocytes, and dermal fibroblasts, and has a well‐defined role in the pathogenesis of pruritus.^[^ ^21^ ^]^ The pro‐oncogenic role of IL‐31 is primarily identified in cutaneous T cell lymphoma.^[^ ^21^ ^]^ Recently, autocrine IL‐31 production by breast cancer cells is found to promote tumor stemness, tumor growth, and metastasis.^[^ ^22^, ^23^ ^]^ Although serum IL‐31 is of less value for diagnosis and prognosis in HCC,^[^ ^24^ ^]^ our unpublished RNA‐sequencing data also determined IL‐31 expression in primary human hepatoma cells but not in primary normal human hepatocytes, suggesting a link between autocrine IL‐31 and HCC. Here, autocrine IL‐31 production by POU2F2^+^ hepatocytes was identified to be required for DEN to induce HCC. And an autocrine signaling loop formed by mutual induction between POU2F2 and IL‐31 was identified to be a decisive event for converting hepatocytes into the origin cells of HCC.

## 2 Results

### 2.1 POU2F2 Promotes HCC Development

**Figure 1:**
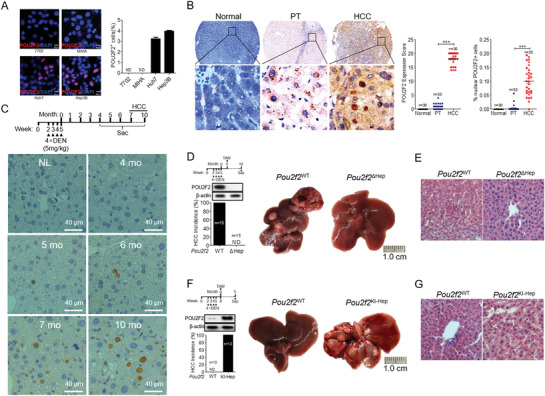
POU2F2 expression is associated with HCC development. A) Immunofluorescence detection of POU2F2^+^ cells in 7702, MIHA, Huh7, and Hep3B cells. Scale bars, 20 µm (left panel). POU2F2^+^ cells are shown as the mean ± SEM (*n* = 3, right panel). ND: no detection. B) Detection of POU2F2 in a human tissue array containing paired distal normal liver tissue (*n* = 30, Normal), paracancerous tissue (*n* = 30, PT), and HCC tissue (*n* = 30, HCC) by IHC assay (left panel). The method for calculating IHC‐determined POU2F2 expression score is detailly described in Supporting Information. The POU2F2 expression score and nuclear POU2F2^+^ cells are shown as the mean ± SEM. Two‐tailed *t*‐test, ∗∗∗ *P* < 0.001 (right panel). C) IHC detection of POU2F2 in liver sections of C57BL/6 mice at months 4, 5, and 6 and tumor sections at months 7 and 10 post‐DEN. The liver of 10‐month‐old C57BL/6 mice without receiving any treatment was used as normal liver (NL). D, left panel) Schematic representation of DEN challenge protocol, immunoblot‐detection of POU2F2 in mouse liver and HCC incidences of *Pou2f2* ^ΔHep^ mice (*n* = 15) and control littermates (*n* = 15, *Pou2f2* ^WT^) at month 10 post‐DEN; ND: no detection. D, right panel) Representative photographs of gross liver appearance of *Pou2f2* ^WT^ and *Pou2f2* ^ΔHep^ mice, and E) representative H&E staining images (200×) of tumor section of *Pou2f2* ^WT^ mouse and liver section of *Pou2f2* ^ΔHep^ mouse at month 10. F, left panel) Schematic representation of DEN challenge protocol, immunoblot‐detection of POU2F2 in mouse liver and HCC incidences of *Pou2f2* ^KI‐Hep^ mice (*n* = 12) and control littermates (*n* = 10, *Pou2f2* ^WT^) at month 5 post‐TAM; ND: no detection. F, right panel) Representative photographs of gross liver appearance of *Pou2f2* ^WT^ and *Pou2f2* ^KI‐Hep^ mice, and G) representative H&E staining images (200×) of liver section of *Pou2f2* ^WT^ mouse and tumor section of *Pou2f2* ^KI‐Hep^ mouse at 5 months post‐TAM.

Compared with two normal human liver cell lines (7702 and MIHA), POU2F2 mRNA was significantly increased in two human HCC cell lines (Hep3B and Huh7) (Figure S1A, Supporting Information), but this protein was detected only in HCC cells (**Figure** **1A**). Immunofluorescence assay determined that ≈4% of Hep3B and ≈3.5% of Huh7 cells expressed POU2F2 and POU2F2 was localized only to the nucleus in the two HCC cell types (Figure 1A). The clinical significance of POU2F2 expression was determined in a human tissue array by immunohistochemistry (IHC) assay. POU2F2 expression was not detected in distal normal liver tissue (0/30), but could be detected in 100% of HCC tissues (30/30) and 70% of paracancerous tissues (21/30) (Figure 1B). POU2F2 localized to the cytoplasm and nucleus in tumor tissues, but was mainly found in the cytoplasm in paracancerous tissues (Figure 1B). The expression scores of POU2F2 and the ratio of nuclear POU2F2^+^ cells were significantly increased in tumor tissues compared with paracancerous tissues (Figure 1B). These data demonstrate a relationship between POU2F2 expression and HCC.

DEN administration induces mice to develop HCC, whose development course is similar to human HCC, by inducing hepatocyte DNA damage.^[^ ^19^ ^]^ POU2F2 expression was identified in mouse liver as early as 5 months after DEN challenge, and then gradually increased in the following months (Figure 1C and Figure S1B,C, Supporting Information). Precisely, ≈0.025% or ≈0.09% of mouse hepatocytes expressed POU2F2 at months 5 or 6 when HCC had not been induced, since H&E staining determined normal liver histology with typically distributed and well‐organized cells in the liver (Figure S1D, Supporting Information). This ratio reached ≈0.9% or ≈1.2% at months 7 or 10 when HCC had been induced, demonstrating that POU2F2 expression is linked to the course of HCC development (Figure S1C, Supporting Information). DEN‐induced POU2F2 was localized mainly to the nucleus of hepatocytes (Figure 1C). In Hep1‐6 cells, a mouse HCC cell line, ≈2% of cells expressed POU2F2, and this protein was localized only in the nucleus (Figure S1E, Supporting Information).

Mice with hepatocyte‐specific knockout (termed *Pou2f2* ^ΔHep^) or knockin (termed *Pou2f2* ^KI‐Hep^) of *Pou2f2* were produced to determine the role of POU2F2 in HCC development. *Pou2f2* ^ΔHep^ mice were intraperitoneally (i.p.) injected with tamoxifen (TAM) daily for 5 consecutive days starting 4 months after DEN challenge to specifically delete hepatic *Pou2f2* (Figure 1D). As shown in Figure 1D, all control littermates developed HCC at 10 months post‐DEN, but none of the *Pou2f2* ^ΔHep^ mice did. The tumor tissue of control littermates showed thin cords of liver cells, resembling human HCC tissue micromorphology,^[^ ^25^ ^]^ whereas *Pou2f2* ^ΔHep^ mice displayed normal liver parenchyma morphology with neat and order hepatic cords (Figure 1E). POU2F2^+^ hepatocytes could not be identified in *Pou2f2* ^ΔHep^ mice, but ≈1.3% of hepatocytes expressed POU2F2 in control littermates at month 10 (Figure S1F, Supporting Information). Immunoblot assay showed POU2F2 expression in the liver of control littermates, but not *Pou2f2* ^ΔHep^ mice, at month 10, validating the results of flow cytometry (Figure 1D). Conversely, *Pou2f2* ^KI‐Hep^ mice were i.p. injected with TAM daily for 5 consecutive days starting 1 week after DEN challenge to specifically induce *Pou2f2* overexpression in hepatocytes (Figure 1F). As shown in Figure 1F, all *Pou2f2* ^KI‐Hep^ mice developed HCC at month 5 post‐TAM, but none of the control littermates did. H&E staining determined thin cords of liver cells in tumor tissue of *Pou2f2* ^KI‐Hep^ mice and normal liver histology of control littermates (Figure 1G). Up to 25% of hepatocytes expressed POU2F2 in *Pou2f2* ^KI‐Hep^ mouse, and only ≈0.025% of hepatocytes expressed POU2F2 in control littermates at this time point (Figure S1G, Supporting Information). Immunoblot assay also determined dramatical expression of POU2F2 in the livers of *Pou2f2* ^KI‐Hep^ mice in comparison with that of control littermates at month 5 post‐TAM, validating the results of flow cytometry (Figure 1F). The 6‐week‐old *Pou2f2* ^KI‐Hep^ mice were injected with TAM daily for 5 consecutive days to induce POU2F2 expression in mouse liver and then were raised normally without any treatment. Liver cancer could not be observed in 24‐month‐old *Pou2f2* ^KI‐Hep^ mice just treated with TAM (data not shown). Therefore, POU2F2 is required for DEN to induce HCC but cannot induce hepatocarcinogenesis by itself.

### 2.2 POU2F2^+^ Hepatocytes Progress to LCSCs during Hepatocarcinogenesis

A promoter‐reporter strategy was used for isolating POU2F2^+^ cells. Briefly, a lentivirus expressing green fluorescent protein (GFP), which was driven by human POU2F2 promoter (Lv‐P_hPOU2F2_‐GFP), was used to infect Hep3B and Huh7 cells for 3 days. GFP expression was observed in both cell lines infected with Lv‐P_hPOU2F2_‐GFP (Figure S2A, Supporting Information). Precisely, ≈4.1% of Hep3B and ≈3.5% of Huh7 cells expressed GFP (Figure S2B, Supporting Information). POU2F2 protein was identified only in GFP^+^ Hep3B and Huh7 cells and not in GFP^−^ cells, suggesting that GFP expression indicates endogenous POU2F2 expression (Figure S2C, Supporting Information). The mRNA levels of eight CSCs‐related markers (including KLF4, OCT4, SOX2, NANOG, EPCAM, CD133, MYC, and ABCB5) were higher in GFP^+^ Hep3B and Huh7 cells than in GFP^−^ cells (Figure S2D, Supporting Information). The protein levels of OCT4, SOX2, and NANOG were also higher in GFP^+^ Hep3B and Huh7 cells than in GFP^−^ cells; however, the differentiation marker albumin was detected only in GFP^−^ Hep3B and Huh7 cells, not in GFP^+^ cells (Figure S2C, Supporting Information). GFP^+^ Hep3B and Huh7 cells formed larger and more spheres than GFP^−^ cells (Figure S2E, Supporting Information). Therefore, POU2F2^+^ hepatoma cells are LCSCs in HCC.

**Figure 2:**
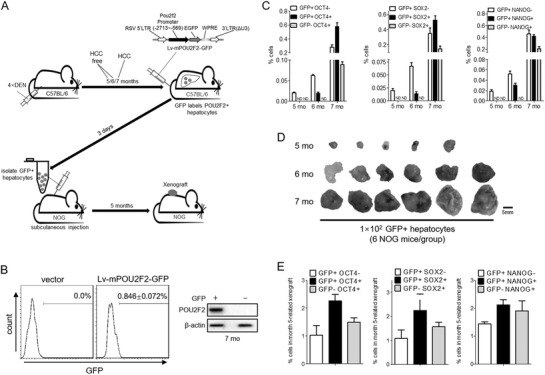
POU2F2^+^ hepatocytes possess CSCs‐like property. A) A lentivirus expressing GFP, which was driven by mouse *Pou2f2* promoter (Lv‐P_mPOU2F2_‐GFP), was used to infect mice by hydrodynamic tail vein injection for 3 days starting 5 or 6 or 7 months after DNE challenge. GFP^+^ hepatocytes (namely POU2F2^+^ hepatocytes) were isolated 3 days after Lv‐P_mPOU2F2_‐GFP infection and then were subcutaneously injected into NOG mice (1 × 10^2^ cells per mouse) for 5 months, followed by xenograft evaluation. B) Mouse hepatocytes were labeled with GFP at month 7 post‐DEN as in (A), followed by flow cytometry detection of GFP^+^ mouse hepatocytes (left panel) and immunoblot detection of POU2F2 in GFP^+^ and GFP^−^ hepatocytes (right panel). C) Labeling mouse hepatocytes at months 5, 6 and 7 post‐DEN with GFP as in (A). Detection of the indicated hepatocytes in mouse liver by flow cytometry. The data are mean ± SEM, *n* = 3. ND: no detection. The representative images of flow cytometry are shown in Figure S3D, Supporting Information. D) GFP^+^ mouse hepatocytes, isolated at months 5, 6, and 7, were transplanted into NOG mice, followed by detection of they‐formed xenograft tumors 5 months later. E) The cells in the fifth month‐related xenografts were infected with Lv‐P_mPOU2F2_‐GFP for 3 days, followed by detection of indicated cells by flow cytometry. The data are mean ± SEM, *n* = 3.

Given that POU2F2 expression occurred before DEN‐induced HCC and POU2F2^+^ hepatoma cells were a type of LCSCs, we hypothesized that POU2F2^+^ hepatocytes might progress into LCSCs which then give rise to HCC. To validate our prediction, the promoter‐reporter strategy was used to isolate GFP^+^ or GFP^−^ hepatocytes (namely POU2F2^+^ or POU2F2^−^ hepatocytes) from DEN‐challenged mice at months 5 and 6 when HCC had not been induced or at month 7 when HCC had been induced (**Figure** **2A**). Month 7‐related GFP^+^ and GFP^−^ hepatocytes were isolated and then immunoblot assay identified POU2F2 only in GFP^+^, but not GFP^−^, hepatocytes (Figure 2B). To determine whether POU2F2^+^ hepatocytes acquire stemness during hepatocarcinogenesis, the fold changes of 84 CSCs‐related genes between GFP^+^ and GFP^−^ hepatocytes were measured by a PCR array. No gene significantly altered its expression at month 5; but five (including OCT4, SOX2, and NANOG) or eight genes were significantly up‐ or down‐regulated at month 6, and 39 or 16 genes were significantly up‐ or down‐regulated at month 7 (Figure S3A,B, Supporting Information). Gene ontology (GO) analysis was performed on the up‐ and down‐regulated genes at month 7. The up‐regulated genes were related to the GO terms including suppression of cell differentiation and cell death, promotion of cell proliferation, and stem cell maintenance (Figure S3C, Supporting Information). The down‐regulated genes were not enriched in any GO term. GFP^+^ OCT4^+^, GFP^+^ SOX2^+^, and GFP^+^ NANOG^+^ hepatocytes were identified at month 6 and reached higher levels at month 7 (Figure 2C and Figure S3D, Supporting Information). These data suggest that POU2F2^+^ hepatocytes have acquired stemness prior to HCC development, but more and stronger expressions of CSCs‐related genes will enhance their stemness during hepatocarcinogenesis.

GFP^+^ hepatocytes (1 × 10^2^ cells per mouse) were transplanted into NOG mice for determining their tumorigenic potential. As shown in Figure 2D, all mice transplanted with month 6 or 7‐related GFP^+^ hepatocytes developed xenografts (*n* = 6 per group), and the tumor size of month 7‐related xenografts was larger than that of month 6‐related xenografts, demonstrating that POU2F2^+^ hepatocytes have stronger tumorigenic potential at month 7 than 6. Surprisingly, month 5‐related GFP^+^ hepatocytes that had not acquired stemness also caused five of six NOG mice to develop xenografts (termed “month 5‐related xenograft”), whose tumor size was smaller than month 6 or 7‐related xenografts’ size (Figure 2D). OCT4^+^ or SOX2^+^ or NANOG^+^ cells were identified in month 5‐related xenografts whose POU2F2^+^ cells had a similar expression profile as month 7‐related GFP^+^ hepatocytes (Figure 2E and Figure S3A, Supporting Information). Thus, POU2F2^+^ hepatocytes might have acquired tumorigenic potential before stemness acquisition, but they will progress into LCSCs by expressing CSCs‐related genes that enhance their tumorigenic property and make them resist cell death and promote cell proliferation.

### 2.3 POU2F2 Converts Hepatocytes to LCSCs by Transactivating NANOG Expression Alone or Synergizing with OCT4 and SOX2 to Transactivate NANOG

**Figure 3:**
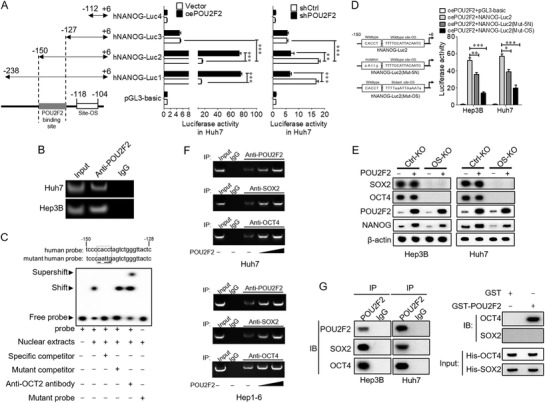
POU2F2 transactivates NANOG expression. A) Huh7 cells were transfected with four human NANOG promoter luciferase reporters (hNANOG‐Luc1/2/3/4) illustrated together with POU2F2 shRNA (shPOU2F2) or POU2F2‐expressing plasmid (oePOU2F2). The control shRNA (shCtrl) or plasmid vector (vector) was used as a control. Cells were lysed 2 days later for analysis of the luciferase activities. The firefly luciferase activity expressed by the pGL3‐basic control vector was arbitrarily defined as 1. The data are mean ± SEM, *n* = 3. Two‐tailed *t*‐test, ∗∗ *P* < 0.01, ∗∗∗ *P* < 0.001. B) ChIP analysis probing POU2F2 recruitment to the NANOG promoter in Hep3B and Huh7 cells. C) Detection of the interaction between POU2F2 and the ≈−150–−128 sequence of human NANOG promoter by EMSA. Nuclear extracts were isolated from Huh7 cells. The specific competitor used was the nonlabeled probe. An anti‐POU2F2 antibody was used for the super‐shift assay. D) Huh7 cells were transfected with hNANOG‐Luc2 or its mutant with mutated site‐OS (hNANOG‐Luc2(Mut‐OS)) or mutated CACCT sequence (hNANOG‐Luc2(Mut‐5N)) illustrated together with POU2F2‐expressing plasmid (oePOU2F2), followed by analysis of luciferase activities 2 days later. The luciferase activity of pGL3‐basic control vector was arbitrarily defined as 1. The data are mean ± SEM, *n* = 3. Two‐tailed *t*‐test, ∗ *P* < 0.05, ∗∗ *P* < 0.01, ∗∗∗ *P* < 0.001. E) Detection of indicated proteins by immunoblot assay in Hep3B/Huh7(OS‐KO) cells transfected with POU2F2‐expressing plasmid (+) or control vector (‐). F) ChIP analysis probing POU2F2 or SOX2 or OCT4 recruitment to the NANOG promoter in Huh7 (upper panel) or Hep1‐6 (lower panel) cells in response to gradual increases in POU2F2. G) Detection of the interaction between OCT4 or SOX2 with POU2F2 by coimmunoprecipitation with an anti‐POU2F2 antibody or control antibody (IgG) in Hep3B and Huh7 cells (left panel). Detection of the interaction between GST‐POU2F2 and His‐SOX2 or His‐OCT4 by GST‐pull‐down (right panel).

As shown in Figure S4A,B, Supporting Information, POU2F2 was required for DEN to induce the expression of CSCs‐related markers and the development of OCT4^+^, SOX2^+^, and NANOG^+^ hepatocytes in mice. It also positively regulated the expression of OCT4, SOX2, and NANOG in human HCC cell lines (Figure S4C,D, Supporting Information). We believe that POU2F2, as a TF, might promote HCC stemness by transactivating the expression of CSCs‐related genes such as OCT4, SOX2, and NANOG. As shown in Figure S5A, Supporting Information and **Figure** **3A**, although POU2F2 overexpression did not activate human OCT4 or SOX2 promoter, the inducing or silencing of POU2F2 significantly increased or reduced the activity of human NANOG promoter (≈−238–+6)‐firefly luciferase reporter in Huh7 cells. Chromatin immunoprecipitation (ChIP) performed on Hep3B and Huh7 cells showed POU2F2 recruitment at NANOG promoter (≈−238–+6) (Figure 3B), confirming our prediction. To identify POU2F2‐binding sequence, human NANOG promoter was truncated. Luciferase reporter assay determined that the sequence ≈−150–−128 located upstream of OCT4‐SOX2 complex‐binding site (site‐OS, ≈−118–−104) was required for POU2F2 to transactivate human NANOG (Figure 3A). Although the ≈−150–−128 sequence does not contain the octamer sequence (ATGCAAAT) for POU family members to interact, it contains a five‐nucleotide sequence (CACCT) that was also identified on upstream (≈−198–−181) of site‐OS (≈−180–−166) on mouse *Nanog* promoter (Figure S5B, Supporting Information). We employed electrophoretic mobility‐shift assay (EMSA) to validate that the CACCT sequence was required for POU2F2 to interact with the ≈−150–−128 or ≈−198–−181 sequence on human or mouse NANOG promoter. As shown in Figure 3C and Figure S5C, Supporting Information, the nuclear extracts of Huh7 or Hep1‐6 cells caused the band shift of the probe containing the CACCT sequence but not the mutant probe containing a mutant sequence. The binding could be abolished by the specific competitor but not the mutant competitor, and the band shift could be super‐shifted by an anti‐POU2F2 antibody. The CACCT sequence was also required for POU2F2 to transactivate human and mouse NANOG since its mutation significantly reduced POU2F2 overexpression‐activated NANOG promoter‐luciferase activity in Hep3B, Huh7, and Hep1‐6 cells (Figure 3D and Figure S5D, Supporting Information).

As shown in Figure 3D and Figure S5D, Supporting Information, POU2F2 overexpression activated human and mouse NANOG promoter‐luciferase activity even though site‐OS was mutated. NANOG protein was reduced, but not absent, in stable Hep3B and Huh7 cells (termed Hep3B/Huh7(OS‐KO)) with a knockout of both OCT4 and SOX2 (Figure 3E). POU2F2 overexpression activated NANOG promoter‐luciferase activity and increased NANOG mRNA and protein levels in Hep3B/Huh7(OS‐KO) cells (Figure 3E and Figure S5E, Supporting Information). Given that NANOG is a core pluripotent TF that induces tumor stemness,^[^ ^6^ ^]^ POU2F2 induces NANOG expression alone may initiate POU2F2^+^ hepatocytes to acquire stemness. Both OCT4 and SOX2 knockout or site‐OS mutation reduced POU2F2‐induced human and mouse NANOG, suggesting a synergetic effect of OCT4, SOX2, and POU2F2 on inducting NANOG expression (Figure 3D,E, and Figure S5D, Supporting Information). The inducing of POU2F2 promoted SOX2 and OCT4 interactions with human or mouse NANOG promoter (Figure 3F). GST‐pull‐down assay determined a direct interaction between POU2F2 and OCT4, but not SOX2 (Figure 3G). CO‐IP assay determined that SOX2 and OCT4 were selectively immunoprecipitated by an anti‐POU2F2 antibody in Hep3B, Huh7, and Hep1‐6 cells (Figure 3G and Figure S5F, Supporting Information). Thus, the three TFs may play a synergetic effect on inducing NANOG expression by forming a protein complex POU2F2‐OCT4‐SOX2.

### 2.4 POU2F2 Overrides p53‐Mediated NANOG Suppression by Interacting with NANOG Promoter

Given that CACCT sequence is a part of p53‐binding site (site‐p53) which is required for p53 to inhibit NANOG transcription,^[^ ^10^ ^]^ we believe that POU2F2 overrides p53‐mediated NANOG suppression by competitively interacting with human NANOG promoter. A probe containing a site‐p53 and CACCT sequence was designed according to human NANOG promoter to validate our prediction by EMSA; and the site‐p53 was constructed by two tandem p53‐binding motifs (motif 1 and 2)^[^ ^10^ ^]^ (Figure S5G, Supporting Information). EMSA showed that recombinant GST‐p53 or HIS‐POU2F2, but not GST‐tag, caused band shift of the probe; the band shift could be super‐shifted by an anti‐p53 or anti‐POU2F2 antibody, suggesting that both p53 and POU2F2 directly interacts with the probe (Figure S5G, Supporting Information). When the concentration of GST‐p53 was fixed, scaling up of HIS‐POU2F2 caused its interaction with the probe to gradually increase, whereas the interaction between GST‐p53 and the probe gradually decreased and finally was blocked completely (Figure S5H, Supporting Information). Given that CACCT sequence is separated from two site‐p53 by hundreds of bases on mouse *Nanog* promoter (Figure S5I, Supporting Information),^[^ ^26^ ^]^ we employed ChIP to validate our prediction in mouse. Hep1‐6 cells were co‐transfected with a plasmid encoding mouse p53 and a plasmid encoding mouse POU2F2. As shown in Figure S5J, Supporting Information, scaling up of POU2F2 caused p53 enrichment at *Nanog* promoter to gradually decrease and finally be blocked completely, validating the result in humans.

### 2.5 NANOG Expression Initiates POU2F2^+^ Hepatocyte to Progress into LCSCs

**Figure 4:**
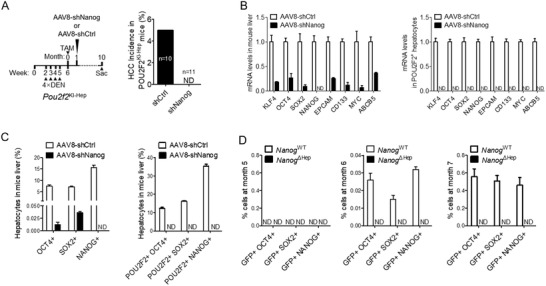
NANOG is required for POU2F2 to induce HCC in DEN‐challenged mice. A) Schematic representation of AAV8‐shNANOG‐mediated *Nanog* knockdown in *Pou2f2* ^KI‐Hep^ mice (*n* = 11, left panel). HCC incidence at month 10 post‐DEN (right panel). AAV8‐control shRNA (*n* = 10, AAV8‐shCtrl) was used as control. ND: no detection. B) Real‐time PCR detection of CSCs‐related markers expression in mouse liver or in POU2F2^+^ mouse hepatocytes. The data are mean ± SEM, *n* = 3. ND: no detection. C) Flow cytometry detection of OCT4^+^ and SOX2^+^ and NANOG^+^ mouse hepatocytes, or POU2F2^+^ OCT4^+^ and POU2F2^+^ SOX2^+^ and POU2F2^+^ MANOG^+^ mouse hepatocytes. The data are mean ± SEM, *n* = 3. ND: no detection. D) *Nanog* ^ΔHep^ mice and their control littermates (*Nanog* ^WT^) were infected with Lv‐P_mPOU2F2_‐GFP as in Figure 2A at months 5, 6, and 7 post‐DEN, followed by detection of indicated mouse hepatocytes. The data are mean ± SEM, *n* = 3. ND: no detection.

To validate our prediction that NANOG initiates POU2F2^+^ hepatocyte to progress into LCSC, DEN‐challenged *Pou2f2* ^KI‐Hep^ mice were infected with AAV8 virus encoding *Nanog* shRNA (AAV8‐shNanog) or control shRNA (AAV8‐shCtrl) starting 1 month after the induction of hepatic *Pou2f2* overexpression (**Figure** **4A**). 10 months after *Pou2f2* overexpression, all *Pou2f2* ^KI‐Hep^ mice infected with AAV8‐shCtrl grew HCC, whereas AAV8‐shNanog infection completely prevented HCC development (Figure 4A). AAV8‐shNanog infection successfully suppressed NANOG expression as shown by non‐detection of hepatic *Nanog* mRNA, NANOG^+^ hepatocytes, and POU2F2^+^ NANOG^+^ hepatocytes at this time point (Figure 4B,C), but it did not affect POU2F2^+^ hepatocytes production (Figure S6A, Supporting Information). The silencing of hepatic NANOG significantly reduced CSCs‐related markers expression in livers or completely suppressed their expression in POU2F2^+^ hepatocytes (Figure 4B). Hepatic NANOG silencing significantly reduced OCT4^+^ or SOX2^+^ hepatocytes production and completely suppressed POU2F2^+^ OCT4^+^ and POU2F2^+^ SOX2^+^ hepatocytes production (Figure 4C).

Next, mice with hepatocyte‐specific knockout of *Nanog* (termed *Nanog* ^ΔHep^) were i.p. injected with TAM starting 4 months post‐DEN to delete hepatic *Nanog*. The hepatocytes of *Nanog* ^ΔHep^ mice and control littermates were labeled with GFP by the promoter‐reporter strategy and extracted at months 5, 6, and 7 post‐DEN. As shown in Figure S6B, Supporting Information, hepatic *Nanog* abrogation did not affect GFP^+^ hepatocytes production at the three time points. GFP^+^ OCT4^+^ or GFP^+^ SOX2^+^ or GFP^+^ NANOG^+^ hepatocytes were induced at month 6, but not month 5, and reached higher levels at month 7 in control littermates, but they could not be induced in *Nanog* ^ΔHep^ mice at the three time points (Figure 4D). The mRNA of CSC‐related markers could not be identified in *Nanog* ^ΔHep^ mice‐derived GFP^+^ hepatocytes at month 7 (Figure S6C, Supporting Information). Thus, both experiments validated our prediction.

### 2.6 POU2F2^+^ Hepatocytes Produce IL‐31 That Conversely Promotes Their Production and Progression into LCSC

**Figure 5:**
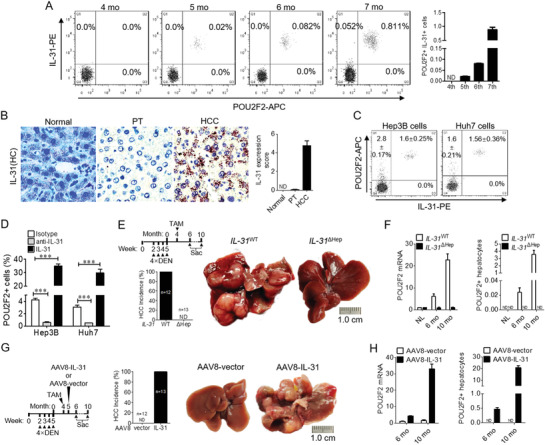
Autocrine IL‐31 production by POU2F2^+^ hepatocytes causes DEN‐induced HCC. A) Detection of POU2F2^+^ IL‐31^+^ hepatocytes in C57BL/6 mice at indicated time points post‐DEN and C) in Hep3B and Huh7 cells by flow cytometry. The data are mean ± SEM, *n* = 3. ND: no detection. B) IHC detection of IL‐31 in distal normal liver tissue (Normal), paracancerous tissue (PT), and HCC tissue (HCC) (left panel). D) Hep3B or Huh7 cells were cultured with a neutralizing anti‐IL‐31 antibody or a recombinant human IL‐31 for 2 days, followed by detection of POU2F2^+^ Hep3B or Huh7 cells flow cytometry. The data are mean ± SEM, *n* = 3. Two‐tailed *t*‐test, ∗∗∗ *P* < 0.001. ND: no detection. E) Schematic representation of hepatic *IL‐31* knock‐out and HCC incidences of *IL‐31* ^ΔHep^ mice (*n* = 13) and control littermates (*n* = 12, *IL‐31* ^WT^) at month 10 post‐DEN; ND: no detection (left panel). Representative photographs of gross liver appearance of *IL‐31* ^ΔHep^ mice and*IL‐31* ^WT^ mice at month 10 post‐DEN (right panel). F) Detection of POU2F2 mRNA or POU2F2^+^ hepatocytes in *IL‐31* ^ΔHep^ and *IL‐31* ^WT^ mice at months 6 and 10 post‐DEN. The livers of 10‐month‐old *IL‐31* ^ΔHep^ and *IL‐31* ^WT^ mice without receiving any treatment were used as normal liver (NL). The data are mean ± SEM, *n* = 3. ND: no detection. G) Schematic representation of *IL‐31* ^ΔHep^ mice infected with AAV8‐IL‐31 (*n* = 13) or AAV8‐vector (*n* = 12) (left panel). HCC incidence (middle panel) and representative photographs of gross liver appearance (right panel) of AAV8‐IL‐31/AAV8‐vector‐infected *IL‐31* ^ΔHep^ mice at month 10 post‐DEN. H) The levels of *Pou2f2* mRNA and POU2F2^+^ hepatocytes in the livers of AAV8‐IL‐31/AAV8‐vector‐infected *IL‐31* ^ΔHep^ mice at months 6 and 10 post‐DEN. The data are mean ± SEM, *n* = 3. ND: no detection.

Primary mouse hepatocytes (PMHs) were isolated at months 4, 5, 6, and 7 post‐DEN, followed by in vitro culture for 3 days. Supernatant IL‐31 could be detected from month 5 and its level gradually increased in the following months, suggesting an autocrine IL‐31 by mouse hepatocytes (Figure S7A, Supporting Information). POU2F2^+^ IL‐31^+^ hepatocytes were also identified in mice at month 5 post‐DEN and then gradually increased in months 6 and 7, demonstrating that POU2F2^+^ mouse hepatocytes produce IL‐31 (**Figure** **5A**). IL‐31 protein and POU2F2^+^ IL‐31^+^ cells were also identified in human HCC tissues and HCC cell lines (Hep3B and Huh7), respectively, validating the findings in mice (Figure 5B,C). A neutralization antibody‐mediated blockade of IL‐31 signaling dramatically decreased the levels of POU2F2^+^ Hep3B or Huh7 cells, whereas recombinant human IL‐31‐mediated activation of IL‐31 signaling had the opposite effect, suggesting that POU2F2^+^ hepatoma cells have acquired autocrine IL‐31 signaling which conversely stimulates their production (Figure 5D).

To validate the roles of IL‐31 on inducing POU2F2 expression and giving rise to HCC, mice with a hepatocyte‐specific knockout of *IL‐31* (termed *IL‐31* ^ΔHep^) were i.p. injected with TAM starting 4 months post‐DEN to specifically delete hepatic *IL‐31*. As shown in Figure 5E, none of the *IL‐31* ^ΔHep^ mice grew HCC at month 10 post‐DEN whereas all control littermates did, suggesting that IL‐31 is required for DEN to induce HCC. Representative histology of liver tissue of *IL‐31* ^ΔHep^ mice or tumor tissue of control littermates is shown in Figure S7B, Supporting Information. *Pou2f2* mRNA was not increased and POU2F2^+^ hepatocytes could not be induced in *IL‐31* ^ΔHep^ mice at months 6 and 10 post‐DEN, suggesting that IL‐31 is required for DEN to induce *Pou2f2* expression (Figure 5F). DEN‐challenged *IL‐31* ^ΔHep^ mice were further infected with AAV8 virus encoding mouse IL‐31 (AAV8‐IL‐31) to restore *IL‐31* expression in hepatocytes starting 1 month after TAM treatment (Figure 5G). AAV8‐IL‐31, but not AAV8‐vector, infection successfully restored IL‐31 expression in the livers of *IL‐31* ^ΔHep^ mice at months 6 and 10 post‐DEN (Figure S7C, Supporting Information). As shown in Figure 5G, Hepatic IL‐31 recovery caused all *IL‐31* ^ΔHep^ mice to develop HCC at month 10 post‐DEN, whereas none of the mice infected with AAV8‐vector developed hepatocarcinogenesis. Representative histology of AAV8‐IL‐31‐induced tumor tissue is shown in Figure S7D, Supporting Information. The infection of AAV8‐IL‐31, but not AAV8‐vector, increased *Pou2f2* mRNA and induced POU2F2^+^ hepatocytes development at months 6 and 10 post‐DEN, suggesting that hepatic IL‐31 recovery restores *Pou2f2* expression (Figure 5H). AAV8‐IL‐31, but not AAV8‐vector, infection also induced the development of POU2F2^+^ OCT4^+^, POU2F2^+^ SOX2^+^, and POU2F2^+^ NANOG^+^ hepatocytes at months 6 and 10 post‐DEN (Figure S7E, Supporting Information). Thus, autocrine IL‐31 signaling is required for POU2F2 expression and POU2F2^+^ hepatocytes progression into LCSCs.

### 2.7 The Mutual Induction between POU2F2 and IL‐31 Forms an Autoregulatory Circuit That Gives Rise to HCC

**Figure 6:**
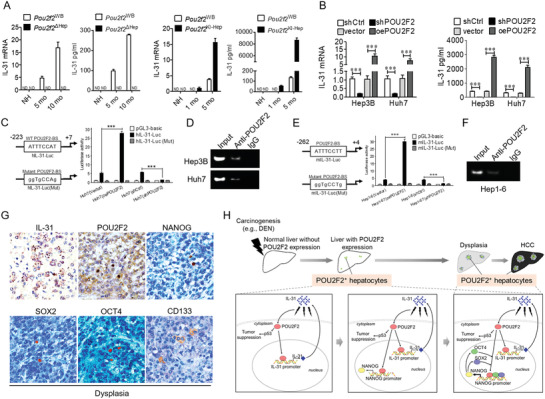
POU2F2‐IL‐31 autoregulatory circuit occurs in cirrhotic tissues. A) PMHs, which were isolated from *Pou2f2* ^ΔHep^/*Pou2f2* ^WT^ mice at months 5 and 10 post‐DEN or from *Pou2f2* ^KI‐Hep^/*Pou2f2* ^WT^ mice at months 1 and 5 post‐DEN, were cultured for 3 days in vitro, followed by detection of supernatant IL‐31 by ELISA and *IL‐31* mRNA by real time PCR. PMHs originated from 10‐month‐old *Pou2f2* ^WT^ mice that grew under normal conditions were used as control normal hepatocytes (NH). The data are mean ± SEM, *n* = 3. ND: no detection. B) The levels of *IL‐31* mRNA (left panel) and supernatant IL‐31 (right panel) of stable Hep3B or Huh7 cells expressing POU2F2 shRNA (shPOU2F2) or a plasmid encoding POU2F2 (oePOU2F2). Control shRNA (shCtrl) or plasmid vector (vector) were used as control. The data are mean ± SEM, *n* = 3. Two‐tailed *t*‐test, ∗∗∗ *P* < 0.001. C) Human *IL‐31* promoter luciferase reporter (hIL‐31‐Luc) and its mutant with mutated POU2F2‐binding site (hIL‐31‐Luc(Mut)) were transfected into stable Huh7 cells over‐expressing POU2F2 (oePOU2F2) or expressing shPOU2F2 for 3 days, followed by detection of luciferase activity. shCtrl and vector were used as control. The data are mean ± SEM, *n* = 3. Two‐tailed *t*‐test, ∗∗∗ *P* < 0.001. D) ChIP analysis probing POU2F2 recruitment to the *IL‐31* promoter in Hep3B and Huh7 cells. E) Mouse *IL‐31* promoter luciferase reporter (mIL‐31‐Luc) and its mutant with mutated POU2F2‐binding site (mIL‐31‐Luc(Mut)) were transfected into stable Hep1‐6 cells over‐expressing POU2F2 (oePOU2F2) or expressing shPOU2F2 for 3 days, followed by detection of luciferase activity. shCtrl and vector were used as control. The data are mean ± SEM, *n* = 3. Two‐tailed *t*‐test, ∗∗∗ *P* < 0.001. F) ChIP analysis probing POU2F2 recruitment to the *IL‐31* promoter in Hep1‐6 cells. G) Detection of IL‐31, POU2F2, NANOG, SOX2, OCT4, and CD133 in tissue array blocks containing human cirrhotic tissues (*n* = 40) by IHC assay. H) Schematic representation of POU2F2‐IL‐31 autoregulatory circuit that controls LCSC and HCC development.

PMHs were isolated from DEN‐challenged *Pou2f2* ^ΔHep^, *Pou2f2* ^KI‐Hep^ mice, and control littermates, followed by in vitro culture for 3 days. Real‐time PCR or ELISA assay determined that *Pou2f2* ^KI‐Hep^ mouse‐originated PMHs expressed more IL‐31 mRNA or protein than control littermate‐originated hepatocytes, and *Pou2f2* ^ΔHep^ mouse‐originated hepatocytes did not express IL‐31, indicating that POU2F2 positively regulates IL‐31 expression (**Figure** **6A**). The silencing of POU2F2 significantly reduced the mRNA and protein levels of IL‐31 in Hep3B and Huh7 cells whereas the inducing of POU2F2 had the opposite effects, validating the findings in mice (Figure 6B). The octamer sequence for POU family members binding was identified in human and mouse *IL‐31* promoters (Figure S8A, Supporting Information). The inducing or silencing of POU2F2 activated or inactivated *IL‐31* promoters in Huh7 and Hep1‐6 cells transfected with the promoter‐firefly luciferase constructs, but mutation of POU2F2‐binding site impaired the activities of human and mouse *IL‐31* promoters (Figure 6C,E). ChIP assay performed on Hep3B, Huh7, and Hep1‐6 cells confirmed POU2F2 enrichment at human and mouse *IL‐31* promoters (Figure 6D,F). Thus, POU2F2 and IL‐31 form an autoregulatory circuit by mutual induction in hepatocytes. Last, the autoregulatory circuit and the corresponding activated CSCs‐related markers (including NANOG, SOX2, OCT4, and CD133) also occurred in human cirrhotic tissues (*n* = 40) which have been suspected to progress into HCC (Figure 6G). Precisely, co‐expression of IL‐31 and POU2F2 was present in 70% of cases; co‐expression of IL‐31, POU2F2, and NANOG was present in 40% of cases; co‐expression of IL‐31, POU2F2, NANOG, and any of the other three indexes was present in ≈15–32.5% of cases (Figure S8B, Supporting Information).

## 3 Conclusion and Discussion

The roles and mechanisms of LCSCs in promoting HCC development have been largely elucidated, although they are not bona fide cells of origin for HCC. Here, carcinogenesis stimuli, for example, DEN, contribute to POU2F2 expression in hepatocytes. POU2F2‐induced NANOG caused hepatocytes to acquire stemness and then be converted to LCSCs by induction of CSCs‐related genes expression (Figure 6H). The inoculation of a given cell population into immunodeficient mice, for example, NOG, in numbers sufficiently low to measure their tumorigenic ability is a common method to analyze the presence of CSCs.^[^ ^7^, ^20^ ^]^ NOG mouse has no lymphocytes or NK cells and has impaired dendritic cells and macrophage function. They can accept a graft consisting of 100 cervical cancer cells and even one melanoma cell, causing they to be sensitive enough to identify CSCs including LCSCs.^[^ ^25^, ^27^, ^28^, ^29^ ^]^ Here, we identified that 100 POU2F2^+^ mouse hepatocytes isolated at months 6 and 7 formed xenograft tumors in NOG mice, suggesting that they are a type of LCSCs with strong tumorigenic ability. Surprisingly, POU2F2^+^ hepatocytes formed xenograft tumors before acquiring stemness, suggesting that POU2F2 expression is a decisive event for converting fully differentiated hepatocytes into the cells of origin for HCC. Nonetheless, stemness acquisition is still required for POU2F2^+^ hepatocytes to initiate hepatocarcinogenesis since hepatic *Nanog* deletion blocked POU2F2^+^ hepatocytes to acquire stemness and finally made these cells lose tumorigenic capacity. In addition, POU2F2 mainly localizes to cytoplasm in paracancerous tissues but localized to both nucleus and cytoplasm in HCC tissues. How this cytosolic‐nuclear shuttling happens in HCC tissues and is suppressed in paracancerous tissues are still unclear. Elucidation of these questions by ongoing experiments is significant for understanding carcinogenesis of POU2F2 in HCC.

POU2F2‐induced NANOG initiated hepatocytes stemness acquisition and malignant transformation. The stem cell state provides a more permissive context for oncogenic transformation by allowing the sequential accumulation of genetic or epigenetic mutations required for oncogenesis.^[^ ^6^ ^]^ Stem cell signals are also integrally linked to tumor initiation and propagation in many types of tumors.^[^ ^6^ ^]^ As a core pluripotency TF, NANOG induces and maintains tumor stemness by promoting the expression of many CSCs‐associated genes including OCT4 and SOX2.^[^ ^6^ ^]^ NANOG also metabolically reprograms tumor‐initiating stem‐like cells (TICs) to contribute to hepatocarcinogenesis, since it suppresses mitochondrial oxidative phosphorylation, as well as ROS generation, and activates fatty acid oxidation to support the self‐renewal and drug resistance of TICs.^[^ ^28^ ^]^ Given that CSCs need to possess unique metabolic properties that are compatible with oncogenic transformation,^[^ ^2^ ^]^ POU2F2‐induced NANOG not only makes hepatocytes acquire stemness but also metabolically reprograms them to undergo malignant transformation.

p53 plays a key role in preventing differentiated hepatocytes from malignant transformation by activating apoptosis, as well as cycle arrest, and suppressing stem cell signaling.^[^ ^8^, ^30^ ^]^ The up‐regulated genes of month 7 were mainly clustered into the GO terms related to suppression of cell differentiation and cell death, promotion of cell proliferation, and stem cell maintenance. These results demonstrate that p53‐mediated tumor suppression has been overridden in POU2F2^+^ hepatocytes. p53 interacts with NANOG promoter and then suppresses NANOG transcription by blocking OCT4‐SOX2 complex binding to NANOG promoter.^[^ ^10^ ^]^ Here, POU2F2‐binding site on NANOG promoter was identified to overlap with a motif of p53‐binding site, and EMSA showed that POU2F2 could block the interaction between p53 and its binding site at human NANOG promoter. Although POU2F2‐binding site was separated from two p53‐binding sites by hundreds of bases on mouse *Nanog* promoter, ChIP assay also confirmed that POU2F2 blocked p53 enrichment at its two binding sites on mouse *Nanog* promoter. Thereby, POU2F2 blocks the interaction between p53 and NANOG promoter and then transactivates NANOG expression alone or recruits OCT4 and SOX2 to the NANOG promoter to induce more NANOG expression by forming POU2F2‐OCT4‐SOX2 complex. These results partially elucidate how POU2F2 makes hepatocytes override p53‐mediated tumor suppression to acquire stemness and undergo transformation (Figure 6H). Whether POU2F2 directly suppresses p53‐transactivated pro‐apoptotic‐ and cell‐cycle arrest‐related genes will be provided by ongoing experiments.

Here, the pro‐oncogenic role of IL‐31 in hepatocarcinogenesis was identified. This pro‐oncogenic effect is closely related to IL‐31‐induced POU2F2 expression and POU2F2‐mediated stemness acquisition. HcPC‐produced IL‐6 promotes HCC initiation and progression, as well as tumor‐produced IL‐31 promotes progression and metastasis in breast cancer, suggesting the key roles of tissue‐ or tumor‐derived cytokines in tumor initiation, progression, and metastasis.^[^ ^9^, ^22^ ^]^ Our data further validate this point. HCC is a typical inflammation‐associated cancer since most HCC cases occur in patients with chronic inflammatory liver diseases. Although considered a non‐inflammatory liver cancer model, DEN‐induced hepatocarcinogenesis is actually influenced by inflammatory signals such as NF*κ*B and IL‐6, and liver inflammation can be detected in this model.^[^ ^31^, ^32^ ^]^ Although autocrine IL‐6 in both HcPC and foci of altered hepatocytes (FAH) promotes HCC progression and tumorigenic growth,^[^ ^9^ ^]^ DEN challenge first induces IL‐6 in Kupffer cells through NF‐*κ*B activation, and this immune cell‐derived IL‐6 is also required for DEN‐induced hepatocarcinogenesis.^[^ ^31^, ^33^ ^]^ Whether and which immune cells express IL‐31 and whether immune cell‐derived IL‐31 is required for hepatocarcinogenesis will be answered by ongoing experiments. HCC has been suspected to arise from premalignant/dysplastic lesions, but the molecular mechanisms underlying this progression are still unclear. Here, the mutual induction between IL‐31 and POU2F2 formed an autoregulatory circuit that functionally promoted hepatocytes to be converted into LCSCs (Figure 6H). The autoregulatory circuit and its corresponding activated CSCs genes were identified in human premalignant lesions, partially uncovering the molecular mechanisms by which dysplastic lesions progress into malignant tumors. Thereby, the disruption of this circuit might be useful in HCC prevention using pharmacological agents. Prevention is of particular importance in HCC since most HCC cases are refractory to currently available therapeutics. POU2F2 and IL‐31 expressions could be identified in macroscopically normal livers, suggesting their potential for predicting hepatocarcinogenesis. Additionally, given the tumorigenic capacity of the hepatocytes expressing POU2F2 and IL‐31, it is rational to identify this type of hepatocytes during liver transplantation for the prevention of tumor propagation.

## 4 Experimental Section

### Mice

C57BL/6J mice and NOG mice were purchased from Charles River (Beijing, China). *Pou2f2* ^ΔHep^, *Pou2f2* ^KI‐Hep^, *Nanog* ^ΔHep^, and *IL‐31* ^ΔHep^ C57BL/6J mice were produced by Shanghai Model Organisms Center (Shanghai, China). The detailed processes for the production of KO/KI mice are detailed in Supporting Information. All studies were performed on male mice. The mouse‐related experiments were performed following a protocol approved by the Animal Care and Use Committee of Capital Medical University affiliated to Beijing You An Hospital. All animal experiments were performed according to the guidelines and approval of the institutional animal care committee.

### Clinical Samples

A commercial HCC tissue array (Cat.No. HLivH090PG01, ShangHai OUTDO BIOTECH) included 30 paired human HCC tissues, paracancerous tissues, and distal normal liver tissues, and a commercial Liver cirrhosis tissue array (Cat.No. TC0078, Wuhan Bioyeargene Biotechnology Co., Ltd.) included 40 cases of human cirrhosis tissues were used for detection of POU2F2, NANOG, SOX2, OCT4, IL‐31, and CD133.

### Cell Cultures

Two normal human liver cell lines (7702 and MIHA) and two human HCC cell lines (Hep3B and Huh7) were maintained in Dulbecco's modified Eagle's medium containing 10% fetal bovine serum in a humidified incubator at 37 °C with 5% CO_2_. PMHs were isolated from mice with or without DEN challenge. The detailed processes for isolation and culture of PMHs are detailed in Supporting Information.

### Mouse HCC Model

All the mice used for the production of murine HCC models were i.p. injected with DEN (5 mg kg^−1^) on day 14 postnatally and once a week for 3 weeks subsequently. *Pou2f2* ^ΔHep^, *Nanog* ^ΔHep^, and *IL‐31* ^ΔHep^ mice were i.p. injected with TAM starting 4 months after DEN challenge to delete hepatic *Pou2f2*, *Nanog*, and *IL‐31*, respectively. *Pou2f2* ^KI‐Hep^ mice were i.p. injected with TAM to induce *Pou2f2* expression in hepatocytes at week 1 post‐DEN. DEN‐challenged *Pou2f2* ^KI‐Hep^ mice were infected with AAV8‐shNanog or AAV8‐shCtrl starting 1 month after TAM treatment. *IL‐31* ^ΔHep^ mice were infected with AAV8‐IL‐31 or AAV8‐vector starting 1 month after TAM treatment. Each mouse was given 2 × 10^11^ AAV8 particles by hydrodynamic tail vein injection. At the indicated time points, HCC incidences were determined by observation of visible HCC tissues. H&E staining was used to examine normal liver or tumor sections.

### Promoter‐Reporter Strategy and Xenografts

Lv‐P_hPOU2F2_‐GFP was used to infect Hep3B and Huh7 cells for 3 days. Flow cytometry was used to measure and/or isolate GFP^+^ Hep3B or Huh7 cells. Lv‐P_mPOU2F2_‐GFP (1 × 10^8^ lentivirus particles per mouse) was used to infect mice by hydrodynamic tail vein injection for 3 days. GFP^+^ mouse hepatocytes were isolated by flow cytometry, followed by determination of their tumorigenicity in NOG mice as previously described.^[^ ^28^ ^]^ Briefly, 1 × 10^2^ GFP^+^ cells were subcutaneously injected into 8‐week‐old male NOG mice (6 NOG mice per group) and xenografts were assessed 5 months later.

### Quantification and Statistical Analysis

Statistical analysis was performed by GraphPad Prism 5.0 software. All of the data shown in the histograms were the results of at least three independent experiments and are presented as the mean ± SEM. The sample size (n) for each statistical analysis was indicated detailly in figure legends. The differences between two groups were compared using two‐tailed unpaired Student's *t*‐test. Differences between values were considered statistically significant when ∗ *P* < 0.05, ∗∗ *P* < 0.01, and ∗∗∗ *P* < 0.001. To PCR array, data analysis was performed using the 2^−ΔΔC^_T_ method and cluster 3.0 was used to perform hierarchical clustering. TreeView was used to produce heatmap. Significant expression change was defined as ≥twofold change.

The detailed experimental materials and methods are included in the Supporting Information.

## Conflict of Interest

The authors declare no conflict of interest.

## Supporting information

Supporting Information

## Data Availability

Research data are not shared.
