# Supplementary material for: POU2F2‐IL‐31 Autoregulatory Circuit Converts Hepatocytes into the Origin Cells of Hepatocellular Carcinoma
Source: Adv Sci (Weinh). 2021 May 2;8(13):2004683. doi: 10.1002/advs.202004683 (PMC10619474; doi:10.1002/advs.202004683)
Supplement: Supplementary file 1 — Supporting Information [file ADVS-8-2004683-s001.pdf]

© 2021 Wiley-VCH GmbH

ADVANCED SCIENCE
Open Access

Supporting Information

for *Adv. Sci.,* DOI: 10.1002/advs.202004683

POU2F2-IL-31 autoregulatory circuit converts hepatocytes into the origin cells of hepatocellular carcinoma

Chunwang Yuan, Lijun Pang, Wenjing Wang,
Yabo Ouyang, Xianghua Guo, and *Kai Liu*[*]

POU2F2-IL-31 autoregulatory circuit converts hepatocytes into the origin cells of hepatocellular carcinoma

*Chunwang Yuan, Lijun Pang, Wenjing Wang, Yabo Ouyang, Xianghua Guo, Kai Liu**

Dr. C. Yuan, Dr. L. Pang, Dr. W. Wang, Dr. Y. Ouyang, Dr. X. Guo, Dr. K. Liu

Capital Medical University Affiliated to Beijing You An Hospital, Beijing Institute of Hepatology

Beijing, 100069, China

E-mail: liukai@ccmu.edu.cn

Chunwang Yuan and Lijun Pang contributed equally to this work.

EXPERIMENTAL MODEL AND SUBJECT DETAILS

Mice: Wildtype C57BL/6J mice and NOD/Shi-scid/Il2r$^{-/-}$ (NOG) mice were purchased form Charles River (Beijing, China). *Pou2f2*$^{\Delta Hep}$, *Pou2f2*$^{KI\text{-}Hep}$, *Nanog*$^{\Delta Hep}$, and *IL-31*$^{\Delta Hep}$ C57BL/6J mice were produced by Shanghai Model Organisms Center (Shanghai, China), which were detailed in “method details”. All studies were performed in male mice. All the mice lived in a standard 12 h light-dark cycle under the specific-pathogen-free conditions, and freely accessed to water and food. The mouse-related experiments were performed in accordance with a protocol approved by the animal care and use committee of Capital Medical University affiliated to Beijing You An Hospital. All animal experiments were performed according to the guidelines and approval of the institutional animal care committee.

Cell lines: A normal human liver cell line 7702 and two human HCC cell lines (Hep3B, Huh7) were maintained in Dulbecco’s modified Eagle’s medium (DMEM) containing 10% fetal bovine serum (FBS) in a humidified incubator at 37 ºC with 5% CO_2. Primary mouse hepatocytes (PMHs) were isolated from wildtype C57BL/6J mice at indicated timepoints after DEN challenge. The methods for isolation and culture of PMHs were detailed in “method details”.

*DNA plasmids***:** NANOG-Luc1 had been used in our previous study[1]. NANOG-Luc1 was

used as the template for amplifying different lengths of human *NANOG* promoter. The amplicons were digested and cloned into pGL3 luciferase vector to produce the other three luciferase reporters NANOG-Luc 2/3/4. 5’-CCGCTCGAGTATAATGAAGGCTCTATCACCTTAGA-3’ was reverse primer. 5’-CGGGGTACCTCCCCACCTAGTCTGGGTTACTCTGC-3’ was forward primer for producing NANOG-Luc2; 5’-CGGGGTACCTGCAGCTACTTTTGCATTACAATGGC-3’ was forward primer for producing NANOG-Luc3; 5’-CGGGGTACCATTACAATGGCCTTGGTGAGACTGG-3’ was forward primer for producing NANOG-Luc4. The fragment of mouse *Nanog* promoter (-307~-83) was amplified by PCR and then this amplicon was cloned into pGL3 luciferase vector to produce mNANOG-Luc. The forward primer 5’-CGGGGTACCGCCGTGGTTAAAAGATGAATAAAGTGAAAT-3’ and the reverse primer 5’-CCGCTCGAGACAGTTAATCCCACCTGCAG-3’ were used for production of mNANOG-Luc. The sequence of human *IL-31* promoter (-223~+7) was amplified by PCR and then this amplicon was cloned into pGL3 luciferase vector. The forward primer 5’-CGGGGTACCTGTGCCTTCTTGTGAAGTATGTGTGTGTCT-3’ and the reverse primer 5’-CCGCTCGAGCCAGATGTGTTCGCCATGGCTGCCT -3’ were used for production of hIL-31-Luc. The sequence of mouse *IL-31* promoter (-262~+4) was amplified by PCR and then this amplicon was cloned into pGL3 luciferase vector. The forward primer 5’-CGGGGTACCATGCCTTCCTGTGTGGTATGTGTATGCGTT-3’ and the reverse primer 5’-CCGCTCGAGAGCCTGGTGTGTTCGCCATGGCCAC -3’ were used for production of mIL-31-Luc. The mutated luciferase reporters were generated by site-directed mutagenesis using the QuikChange kit (Cat. No 200521, Agilent Technologies).

A gene fragment encoding human POU2F2 ORF amplified from pCDNA3-POU2F2 (our lab) was cloned into Eco R1 and Sma 1 sites of pGEX-3X (Cat.No.28-9546-54, GE Healthcare Life Sciences) to yield pGEX-3X-POU2F2, which encodes an N-terminal GST-tagged human POU2F2. Three gene fragments encoding human OCT4, SOX2, and POU2F2 were amplified from pCDNA3-OCT4 (our lab), pMXs-hSOX2 (Cat. No. 17218, Addgene), and pCDNA3-POU2F2, respectively, followed by clone into Nde I and BamH I sites of pET16b to generate three plasmids pET16b-OCT4, pET16b-SOX2, and pET16b-POU2F2 which encode

N-terminal HIS-tagged human OCT4, SOX2, and POU2F2, respectively. pGEX-human p53-(1-393) was used for producing GST-p53 [2].

Virus vectors: A fragment of mouse (-2713~-569) or human (-3018 to +16) *POU2F2* promoter was subcloned into pRRLSIN.cPPT.PGK/GFP.WPRE (Cat.No. 12252, Addgene) by substitution for PGK promoter to produce a lentiviral vector termed Lv-$P_{mPOU2F2}$-GFP or Lv-$P_{hPOU2F2}$-GFP as described previously [3]. The lentivirus (pRRLSIN.cPPT.ΔPGK/GFP.WPRE) with PGK promoter deletion was used as a lentivirus control. An AAV8 plasmid (AAV8-eGFP, our lab) containing the CMV promoter and the eGFP coding sequence (CDS) was used to produce AAV8-IL-31 plasmid by replacing the eGFP CDS with mouse IL-31 CDS fragment. The plasmid AAV8-sh*NANOG* was produced in a same way by replacing eGFP CDS with mouse *Nanog* shRNA. Helper-Free system was used to prepare AAV8-IL-31 and AAV8-sh*Nanog* as described previously [4]. Viral particles were harvested 48 hours after transfection. CsCl gradient centrifugation was used to purify viral particles, followed by determination of viral titers by real time PCR. AAV8-eGFP was used as a control (termed AAV8-vector).

Primers and probes for TaqMan-based real-time PCR: The primers and probes for detecting mRNA levels of human or mouse genes were obtained from ThermoFisher. POU2F2 (Assay ID, Hs00922172_m1), β-actin (Assay ID, Hs01060665_g1), NANOG (Assay ID, Hs02387400_g1), OCT4(Assay ID, Hs04260367_gH), SOX2 (Assay ID, Hs01053049_s1), CD133 (Assay ID, Hs04975794_g1), EPCAM (Assay ID, Hs00901884_g1), MYC (Assay ID, Hs00905030_m1) and ABCB5 (Assay ID, Hs04188792_m1) were primers and probes for measuring human genes. POU2F2 (Assay ID, Mm00448354_m1), β-actin (Assay ID, Mm02619580_g1), NANOG (Assay ID, Mm02019550_s1), OCT4(Assay ID, Mm03053917_g1), SOX2 (Assay ID, Mm03053810_s1), CD133 (Assay ID, Mm01211402_m1), EPCAM (Assay ID, Mm00493214_m1), MYC (Assay ID, Mm00487804_m1) and ABCB5 (Assay ID, Mm01225815_m1) were primers and probes for measuring mouse genes.

Method details

Production of conditional knockout or knockin mice: *Pou2f2*$^{F/F}$, *Nanog*$^{F/F}$ and *IL-31*$^{F/F}$ mice were generated by Shanghai Model Organisms Center (Shanghai, China). The three models were generated by clustered regularly interspaced short palindromic repeats (CRISPR)/Cas9 technology in C57BL/6J mouse background. To generate *Pou2f2*$^{F/F}$ or Nanog$^{F/F}$ or *IL-31*$^{F/F}$ mice, the exons 2-4 of *Pou2f2* allele or the exon 2 of *Nanog* allele or the exon 3 of *IL-31* allele are flanked by loxp sites. The donor vectors containing flox sites flanking exons 2-4 for *Pou2f2* or flanking exon 2 for *Nanog* or flanking exon 3 for *IL-31* and 2 homology arms were used as a template. The donor vector with gRNAs and Cas9 mRNA was microinjected into C57BL/6J fertilized eggs. The positive founder mice were mated to wildtype C57BL/6J mice to obtain *Pou2f2* or *Nanog* or *IL-31* flox heterozygous mice, which were crossed with B6.129-Alb$^{tm1.1(CreERT2)Smoc}$ mice (Shanghai Model Organisms Center, NM-KI-00002) to generated *Pou2f2*$^{F/F}$/albumin-CreER2 (*Pou2f2*$^{\Delta Hep}$) or *Nanog*$^{F/F}$/albumin-CreER2 (*Nanog*$^{\Delta Hep}$) or *IL-31*$^{F/F}$/albumin-CreER2 (*IL-31*$^{\Delta Hep}$) mice. Correct genotype was determined by PCR analysis.

Pou2f2$^{KI\text{-}Hep}$ mice were also generated by Shanghai Model Organisms Center (Shanghai, China) by CRISPR/Cas9 technology in C57BL/6J mouse background. *Pou2f2* CDS was inserted into a vector which contains a CAG promoter, WPRE 3' UTR and ploy A sequence. A floxed “stop” element were then inserted between *CAG* promoter and *Pou2f2* CDS region to form the pCAG-loxp-stop-loxp-POU2F2-WPRE-pA fragment, followed by cloning the 5' and 3' homologous arms of the ROSA26 locus into this vector to form the targeting vector. The targeting vector was micro-injected into C57BL/6J mouse fertilized eggs with gRNA and Cas9 mRNA. The positive founders were mated to wildtype C57BL/6J mice to obtain homozygous *Pou2f2* knockin (*Pou2f2*-KI$^{F/F}$) mice, which were crossed with B6.129-Alb$^{tm1.1(CreERT2)Smoc}$ mice to generate *Pou2f2*-KI$^{F/F}$/albumin-CreER2 (*Pou2f2*$^{KI\text{-}Hep}$) mice.

Pou2f2$^{\Delta Hep}$ or *Nanog*$^{\Delta Hep}$ or *IL-31*$^{\Delta Hep}$ mice were i.p. injected with 75mg/kg/day Tamoxifen (TAM) (Sigma, T5648) for 5 days to activate Cre and then specifically delete hepatic *Pou2f2* or *Nanog* or *IL-31*. To induce POU2F2 expression in mouse hepatocytes, *Pou2f2*$^{KI\text{-}Hep}$ mice were also i.p. injected with 75mg/kg/day TAM for 5 days. The littermates of *Pou2f2*$^{\Delta Hep}$ or *Nanog*$^{\Delta Hep}$

or *IL-31*$^{\Delta Hep}$ or *Pou2f2*$^{KI-Hep}$ mice were used as wildtype controls (*Pou2f2*^{WT}).

Pou2f2^{KI-Hep} mice were intravenously injected with AAV8-shNanog (2×10^{11}/mouse) to specially suppress hepatic *Nanog* or *IL-31*$^{\Delta Hep}$ mice were intravenously injected with AAV8-IL-31 (2×10^{11}/mouse) to specially induce IL-31 in mouse hepatocytes as previously described [4]. AAV8-shCtrl or AAV8-vector was used as the control of AAV8-shNanog or AAV8-IL-31.

Mouse model: C57BL/6J mice were intraperitoneally (i.p.) injected with DEN (5mg/kg) on day 14 postnatally and once a week for three weeks thereafter. The mice were sacrificed at months 4, 5, 6, 7 and 10 post-DEN. At each timepoint, mice livers were removed and separated into individual lobes, which were fixed with 4% paraformaldehyde overnight and paraffin embedded. The normal liver sections or large tumor (>0.5mm) sections were immunohistochemical stained for determination of POU2F2 expression. Or mice livers were used for isolation of PMHs which were stored in liquid nitrogen until analysed. To KO/KI mice, *Pou2f2*$^{\Delta Hep}$ or *Nanog*$^{\Delta Hep}$ or *IL-31*$^{\Delta Hep}$ mice were challenged with DEN in a previous described way and then were i.p. injected with TAM starting 4 months after DEN challenge to delete hepatic *Pou2f2* or *Nanog* or *IL-31*. *Pou2f2*$^{KI-Hep}$ mice were treated with TAM to induce hepatic *Pou2f2* overexpression at week 1 post-DEN. The liver lobes and large tumors (>0.5 mm) were fixed with 4% paraformaldehyde overnight and paraffin embedded, followed by H&E staining. Primary hepatocytes (PMHs) were also isolated from *Nanog*$^{\Delta Hep}$ or *IL-31*$^{\Delta Hep}$ mice at indicated timepoints and then were stored in liquid nitrogen until analysed.

Detection of IL-31 in the supernatant of PMHs by ELISA: PMHs were obtained from DEN challenged-C57BL/6J mice at indicated timepoints with a two-step perfusion method as previously described [5]. After perfusion, cell suspension was filtered through a 100-μm cell strainer, followed by centrifugation at 60g for 3 minutes at 4°C. PMH cells were washed for 3 times and after the last washing, these cells were suspended in Williams E medium supplemented with 30% FBS and 10% DMSO and then were immediately stored in liquid nitrogen until analysed. Trypan blue exclusion test determined that the viability of cryopreserved PMHs was up to 90%. PMHs were cultured as previously described [5]. Briefly,

PMHs were resuspended in Williams E medium supplemented with 10% FBS, 18 μg/ml hydrocortisone, 100 U/ml penicillin, 100 μg/ml streptomycin, 3 μg/ml insulin and 2 mM L-glutamine. PMHs were plated on collagen-coated cell culture plates and then unattached cells were washed away and fresh media were added 12 hours after plating. Three days after culture, the supernatant of PMHs and the cells themselves were collected. Supernatant IL-31 was detected by an ELISA Kit (Cat.No. BMS6030, ThemoFisher) as manufacturer's instruction.

Production of stable knock down/knock in cell lines and knockout cell lines: A plasmid expressing *POU2F2* shRNA (Cat.No. sc-42554-SH, Santa Cruz) or a plasmid (pIRES-POU2F2) were used to transfect Hep3B and Huh7 cells, followed by selection of stable cell lines expressing *POU2F2* shRNA or human POU2F2 with G418. Hep3B and Huh7 cells were co-transfected with constructs expressing sgRNAs targeting *OCT4* (Cat.No. sc-410951, Santa Cruz) and *SOX2* (Cat.No. A35511, ThermoFisher) to produce Hep3B/Huh7(OS-KO) cells as previously described [6].

Immunoblot analysis: As previously described[1], cells were homogenized in the RIPA buffer (Cat.No ab156034, Abcam) and Pierce™ Rapid Gold BCA Protein Assay Kit (Cat. No A53225, Abcam) was used to measure protein concentration. Proteins (10~50 μg) were separated on 10 or 12 or 15% SDS-PAGE gels, followed by transferring of separated proteins to PVDF membranes. 5% non-fat milk was used to block PVDF membrane. Anti-SOX2 antibody (Cat.No.ab97959, Abcam), anti-OCT4 antibody (Cat.No. ab181557, Abcam), anti-POU2F2 antibody (Cat.No. ab179808, Abcam), anti-GFP antibody (Cat.No. sc-8334, Santa Cruz), anti-albumin antibody(Cat.No. sc-51515, Santa Cruz), anti-NANOG antibody(Cat.No. sc-374001, Santa Cruz), and anti-β-actin antibody (Cat.No. 3700, Cell signaling) were used for the specific primary antibodies.

Immunohistochemical staining (IHC) and immunofluorescence assays: IHC staining was performed on HCC tissue array (Cat.No. HLivH090PG01, ShangHai OUTDO BIOTECH) and

Liver cirrhosis array (Cat.No. TC0078, Wuhan Bioyeargene Biotechnology Co., Ltd) using the streptavidin-biotin peroxidase complex method and the antibodies, including anti-POU2F2 antibody (Cat.No. ab179808, Abcam), Anti-SOX2 antibody (Cat.No.ab97959, Abcam), anti-OCT4 antibody (Cat.No. ab181557, Abcam), anti-NANOG antibody(Cat.No. sc-374001, Santa Cruz), anti-IL-31 antibody (Cat.No. ab102750, Abcam) and anti-CD133 antibody (Cat. No. ab19898, Abcam). The extent of the staining, defined as the percentage of positive staining areas of tumor cells in relation to the whole tumor area, was scored on a scale of 0-5: 0; 1, 1-10%; 2, 11-25%; 3, 12-50%; 4, 51-75%; and 5, 76-100%. Staining intensity was scored on a scale of 0-4: 0, negative; 1, weak; 2, moderate; 3, strong; 4, very strong. The expression score (range 0-20) was calculated by multiplying the positive and intensity scores. Immunofluorescence was used to detect GFP^+ Hep3B and Huh7 cells 3 days after Lv-$P_{hPOU2F2}$-GFP infection as previously described [7].

Flow cytometry and Luciferase reporter assays: Flow cytometry was executed as previously described[1]. Anti-POU2F2-APC antibody (Cat.No. 130-103-699, Miltenyi Biotec) or anti-IL-31-PE (Cat.No. 659603, BioLegend) were used for detection of POU2F2 or IL-31 in Hep3B/Huh7 cells or PMHs. After labeling POU2F2 with GFP in Hep3B/Huh7 cell or in mice using the promoter-reporter strategy, GFP^+ cells were detected by flow cytometry. Lv-$P_{mPOU2F2}$-GFP infected PMHs were stained with anti-OCT4-PE (Cat.No. NB100-2379PE, Novus) or anti-SOX2-PE (Cat.No. 656103, BioLegend) or anti-NANOG-PE (Cat.No. NB100-58842PE, Novus) and then to detect GFP^+ $OCT4^+$ / GFP^+ $SOX2^+$ / GFP^+ $NANOG^+$ PMHs by flow cytometry. The luciferase activity was performed as previously described[1]. Briefly, the plasmid pSV-RL, which expressed the Renilla luciferase under the control of the SV40 promoter, was used as internal control to monitor the transfection efficiency. The Promega dual luciferase assay kit was used to detect the luciferase activities 2 days after transfection.

Electrophoretic mobility shift assay (EMSA): The detailed procedures of EMSA was described previously[1]. Briefly, Nuclear extracts from Huh7 or Hep1-6 cells were prepared

using the Nuclear Extraction Kit (Abcam) according to the manufacturer's instructions. Oligonucleotides for the EMSA were labeled with the Biotin 3' End DNA Labeling Kit (Pierce). TCCCCACCTAGTCTGGGTTACTC and TCCCAATTGAGTCTGGGTTACTC were the nucleotide sequences of the probe and mutant probe for detection of the binding of POU2F2 to human *NANOG* promoter. GGGTCACCTTACAGCTTC and GGGTAATTGTACAGCTTC were the nucleotide sequences of the probe and mutant probe for detection of the binding of POU2F2 to mouse *Nanog* promoter. For supershift assay, anti-POU2F2 antibody (Cat.No. ab179808, Abcam) was added into the nuclear extracts and incubated on ice for 10 minutes prior to the addition of probe. The LightShift Chemiluminescent EMSA Kit (Thermo Fisher) was used to detect the signals of the probe and the bandshifts. GTTTTCTAGTTCCCCACCTAGTCTGGGTTACTC was the nucleotide sequence of the probe for detection of GST-p53 and HIS-POU2F2 to human *NANOG* promoter.

Sphere-formation assay: $GFP^{+/-}$ Hep3B and Huh7 cells were isolated by flow cytometry 3 days after labeling POU2F2 with GFP using the promoter-reporter strategy, followed by sphere formation assay as previously described [1]. Briefly, cells were resuspended in serum-free DMEM/F12 (1:1 ratio) supplemented with 100IU/ml penicillin, 100 μg/ml streptomycin, 20 ng/ml human EGF, 10 ng/ml human FGF, 2% B27 supplement without vitamin A, and 1% N2 supplement. Cells (200 cells/well) were subsequently cultured in the ultra-low attachment plate (Cat.No. 3261, Corning) for one week for the analysis of their self-renewal ability.

PCR array and TaqMan-based real-time PCR: Four RT^2 Profiler PCR Arrays were used for detection of 84 CSCs-related genes (Cat.No. 330231 PAHS-176ZA, QIAGEN) in GFP^+ and GFP^- Hep3B cells according to manufacturer's instructions. The detailed procedures for TaqMan-based real time PCR had been described previously [8]. Total RNA was isolated from cells homogenized in Trizol (Cat.No.15596018, Thermo Fisher), followed by synthesis of cDNA using the SuperScript III First-Strand Synthesis system (Cat.No.18080051, Thermo Fisher). Applied Biosystem 7500 Fast PCR system was used for real time PCR assay. The primers and probes were obtained from ThermoFisher.

Chromatin Immunoprecipitation (ChIP) assay: Detailed procedures for ChIP had been described in our previous study[1]. The antibodies for detecting SOX2 (Cat.No.ab97959, Abcam), OCT4 (Cat.No. ab181557, Abcam) and POU2F2 (Cat.No. ab179808, Abcam) were used for immunoprecipitating the DNA fragments that interacted with the related proteins. The forward and reverse primers for detection of the binding of POU2F2 or OCT4 or SOX2 to human *NANOG* promoter by PCR were as follow: 5'-AAAGTTTTATCCCATTCCTG-3' and 5'-TTAAAATCCTGGAGTCTCT-3' [1]. The forward and reverse primers for detecting the binding of POU2F2 to mouse *Nanog* promoter were as follow: 5'-GCCGTGGTTAAAAGATGAATAAAGTGAAAT-3' and 5'-ATGGACATTGTAATGCAAAAGAAGCTGTAA -3'. The forward and reverse primers for detecting the binding of POU2F2 to human *IL-31* promoter were as follow: 5'-TGTGCCTTCTTGTGAAGTATGTGTGTGTCT-3' and 5'-CCAGATGTGTTCGCCATGGCTGCCT-3'. The forward primer 5'-ATGCCTTCCTGTGTGGTATGTGTATGCGTT-3' and the reverse primer 5'-AGCCTGGTGTGTTCGCCATGGCCAC -3' were used for detecting the binding of POU2F2 to mouse *IL-31* promoter. Two primer pairs for detection of POU2F2 binding to two p53-binding sties at mouse *Nanog* promoter were as follow: 5'-AGGTTTTACAGTGAGAACTTGT-3'/5'-TGAGACAGGTTTTTCTCTGTTG-3' and 5'-CTGAAAGGAAAGCCGTGTATAA-3'/5'-GATCTGAGTTTCCTCTTCCATA-3' [9].

Co-immunoprecipitation assay (CO-IP): As described previously[1], Hep3B/Huh7/Hep1-6-origined cell lysates with 5 mg protein were pre-cleared using protein A/G PLUS-agarose beads (Santa Cruz Biotechnology) and then incubated at 4°C overnight with the anti-POU2F2 antibody(Cat.No. ab179808, Abcam). The immunocomplexes were precipitated with protein A/G agarose beads, followed by immunoblot analysis using anti-SOX2 antibody (Cat.No.ab97959, Abcam) and anti-OCT4 antibody (Cat.No. ab181557, Abcam).

Protein expression in Escherichia coli and GST pull down assays: Detailed procedures for the expression of proteins in *E. coli* and their purification had been described before[1]. Briefly, the *E. coli* BL21 strain was transfected with pGEX-3X-POU2F2, pET16b-SOX2 or pET16b-OCT4 for the expression of GST-POU2F2, His-SOX2 or His-OCT4. Cells were grown at 37 ° C until the OD600 reached 0.7-0.9 and then treated with isopropyl β-D-thiogalactopyranoside (0.2 mM) at 25°C for 2~4 hours for the induction of protein expression. Cells were harvested and lysed by sonication on ice in the lysis buffer containing protease inhibitors. The pierce GST Spin Purification Kit (Cat.No 16106, Thermo Scientific) was then used to purify the recombinant protein GST-POU2F2. The HIS-SOX2 and HIS-OCT4 were purified using Ni-NTA Spin Columns (Cat.No 31014, QIAGEN). GST (400μg) and GST-POU2F2 (400μg) were bound to Glutathione Sepharose 4B beads (Cat.No 17-0756-01, GE Healthcare) by gentle rocking motion on a rotating platform for 4 hours at 4 °C, following by removing of unbound GST/GST-POU2F2 by washing with PBS. HIS-SOX2 (400μg) or HIS-OCT4 (400μg) was mixed with the beads by gentle rocking motion for 4 hours at 4°C, following by excessive washing with PBS. Each sample was loaded on 10% SDS-PAGE and then blotted onto membranes, and the antibodies anti-SOX2 (Cat.No.ab97959, Abcam) and anti-OCT4 (Cat.No. ab181557, Abcam) were used for immunoblotting.

References:

[1] K. Liu, J. Lee, J. Y. Kim, L. Wang, Y. Tian, S. T. Chan, C. Cho, K. Machida, D. Chen, J. J. Ou, *MOL CELL* **2017**, *68*, 281.

[2] A. Ayed, F. A. Mulder, G. S. Yi, Y. Lu, L. E. Kay, C. H. Arrowsmith, *Nat Struct Biol* **2001**, *8*, 756.

[3] J. Shan, J. Shen, L. Liu, F. Xia, C. Xu, G. Duan, Y. Xu, Q. Ma, Z. Yang, Q. Zhang, L. Ma, J. Liu, S. Xu, X. Yan, P. Bie, Y. Cui, X. W. Bian, C. Qian, *HEPATOLOGY* **2012**, *56*, 1004.

[4] L. Vila, I. Elias, C. Roca, A. Ribera, T. Ferre, A. Casellas, R. Lage, S. Franckhauser, F. Bosch, *Mol Ther Methods Clin Dev* **2014**, *1*, 14039.

[5] H. Li, Q. Zhuang, Y. Wang, T. Zhang, J. Zhao, Y. Zhang, J. Zhang, Y. Lin, Q. Yuan, N. Xia, J. Han, *CELL MOL IMMUNOL* **2014**, *11*, 175.

[6] N. Fogarty, A. McCarthy, K. E. Snijders, B. E. Powell, N. Kubikova, P. Blakeley, R. Lea, K. Elder, S. E. Wamaitha, D. Kim, V. Maciulyte, J. Kleinjung, J. S. Kim, D. Wells, L. Vallier, A. Bertero, J. Turner, K. K. Niakan, *NATURE* **2017**, *550*, 67.

[7] K. Liu, Y. Shi, X. H. Guo, Y. B. Ouyang, S. S. Wang, D. J. Liu, A. N. Wang, N. Li, D. X. Chen,

CELL DEATH DIS **2014**, *5*, e1078.

[8] L. Pang, L. Xu, C. Yuan, X. Li, X. Zhang, W. Wang, X. Guo, Y. Ouyang, L. Qiao, Z. Wang, K. Liu, *Mol Carcinog* **2019**, *58*, 2118.

[9] T. Lin, C. Chao, S. Saito, S. J. Mazur, M. E. Murphy, E. Appella, Y. Xu, *NAT CELL BIOL* **2005**, *7*, 165.

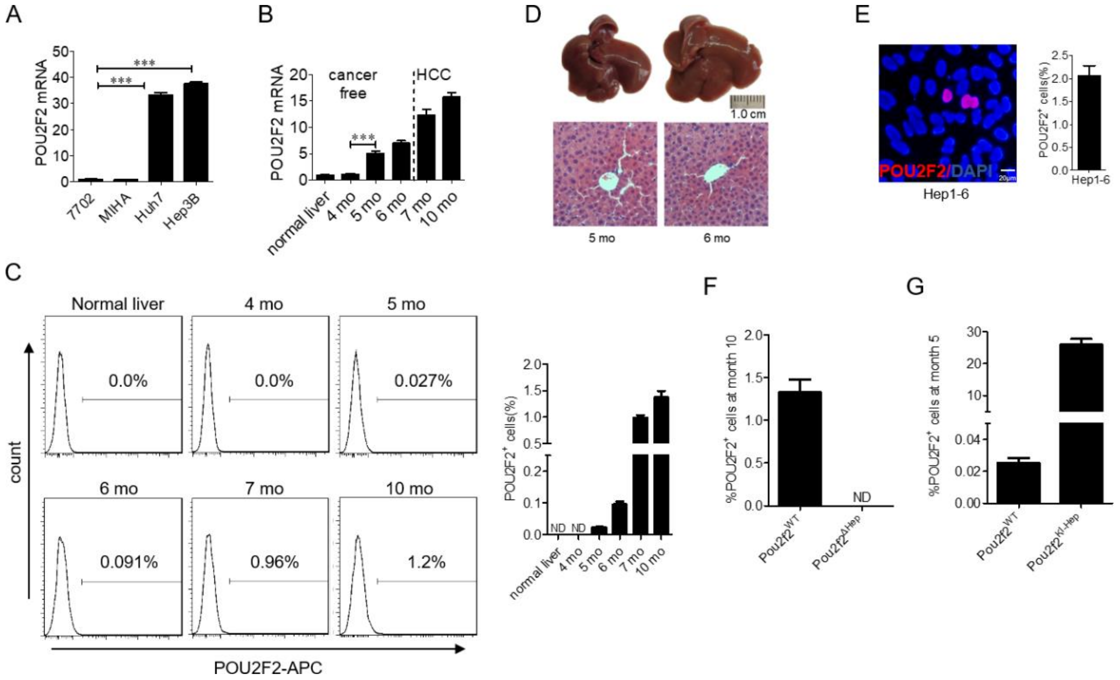

Figure S1. Related to Figure 1

(A) Detection of *POU2F2* mRNA in 7702, MIHA, Huh7 and Hep3B cells by real-time PCR. The data are mean ± SEM, n=3. Two-tailed *t*-test, ∗∗∗ *P*<0.001.

(B, C) Mice were treated as in Figure 1C. Detection of *Pou2f2* mRNA (B) and $POU2F2^{+}$ hepatocytes (C) in mice livers at months 4, 5, 6, 7, and 10 post-DEN. The liver of ten-month-old mice without receiving any treatment was used as normal liver. The data are mean ± SEM, n=3. Two-tailed *t*-test, ∗∗∗ *P*<0.001. ND: no detection.

(D) Representative photographs of gross liver appearance of C57B/L6J mice (upper panel), and representative H&E staining images (200×) of liver section of mice at month 10 post-DEN (lower panel).

(E) Immunofluorescence detection of POU2F2 in Hep1-6 cells.

(F, G) Detection of POU2F2^{+} hepatocytes by flow cytometry in *Pou2f2*$^{\Delta Hep}$ mice at month 10 post-DEN (F) and in DEN-challenged *Pou2f2*$^{KI\text{-}Hep}$ mice at month 5 post-TAM (G). The littermates (*Pou2f2*^{WT}) were used as control. The data are mean ± SEM, n=3. ND: no detection.

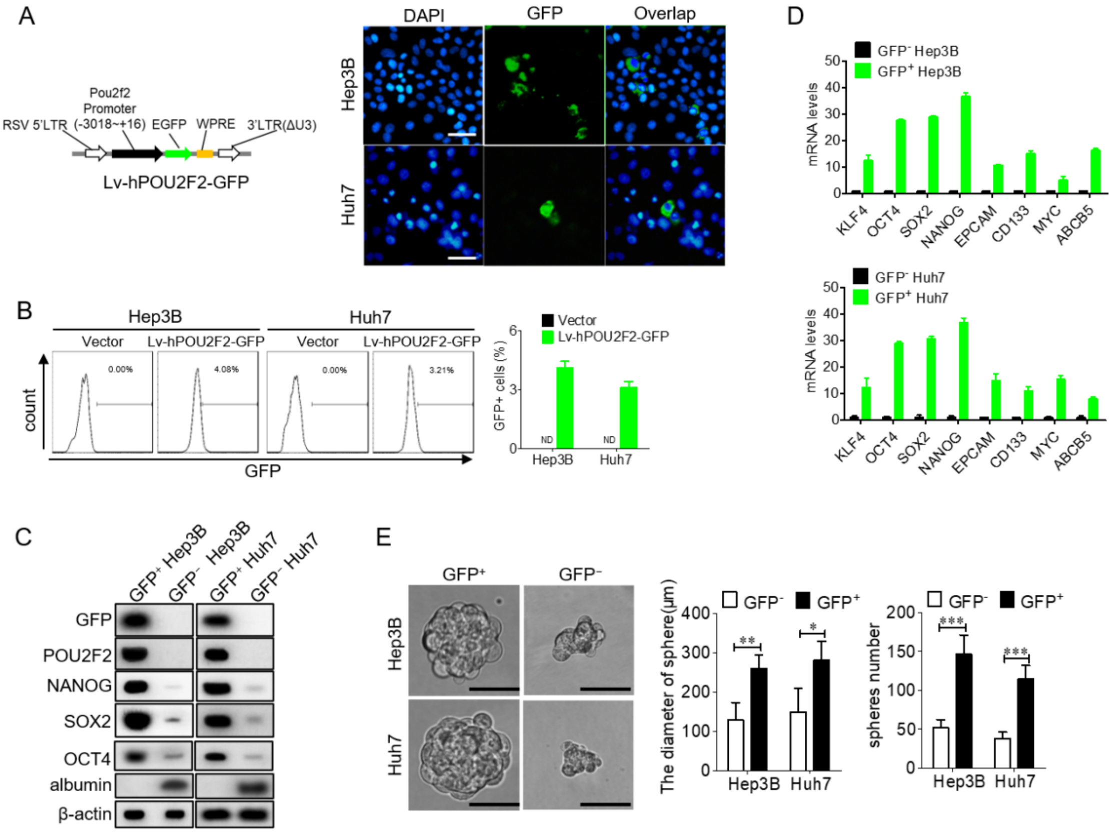

Figure S2. POU2F2^{+} hepatoma cells possess CSC characteristics. Related to Figure 2.

(A, B) The schematic construct of Lv-P$_{hPOU2F2}$-GFP (A, left panel). Hep3B and Huh7 cells were infected with Lv-P$_{hPOU2F2}$-GFP. Three days later, GFP^{+} Hep3B and Huh7 cells were detected by immunofluorescence (A, right panel) and flow cytometry (B). The data are mean ± SEM, n=3. ND: no detection.

(C-E) GFP^{+} and GFP^{-} cells were isolated from Hep3B or Huh7 cells 3 days after Lv-P$_{POU2F2}$-GFP infection. Detection of indicated proteins by immunoblot (C) or the fold changes of 8 CSCs-related markers by real-time PCR (D) in GFP^{+} and GFP^{-} cells. The mRNA level of each CSCs-related marker in GFP^{-} cells was defined as 1 arbitrarily. Detection of self-renewal of GFP^{+} or GFP^{-} cells by sphere-formation assay (E). The data are mean ± SEM, n=3. Two-tailed *t*-test, ∗ P<0.05, ∗∗ P<0.01, ∗∗∗ P<0.001.

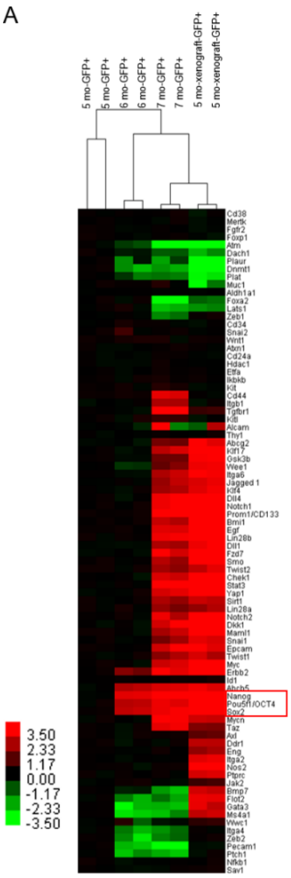

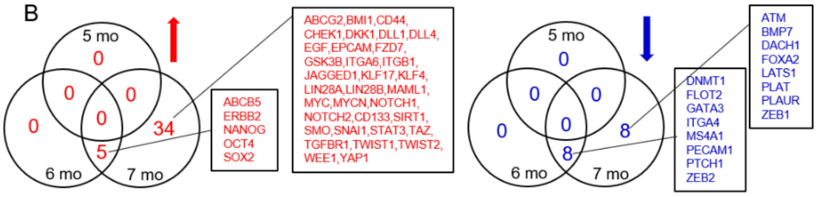

C

Gene Ontology Term	*P*-Value
negative regulation of cell differentiation	1.55E-10
regulation of cell proliferation	5.25E-08
cell fate commitment	6.76E-08
positive regulation of cell proliferation	9.76E-07
Notch signaling pathway	1.04E-05
regulation of transcription from RNA polymerase II promoter	1.34E-05
embryonic morphogenesis	1.37E-05
regulation of transcription, DNA-dependent	2.02E-05
regulation of RNA metabolic process	2.65E-05
regulation of transcription	3.12E-05
negative regulation of apoptosis	3.44E-05
negative regulation of programmed cell death	3.76E-05
stem cell maintenance	3.82E-05
negative regulation of cell death	3.82E-05
negative regulation of gene expression	4.23E-05

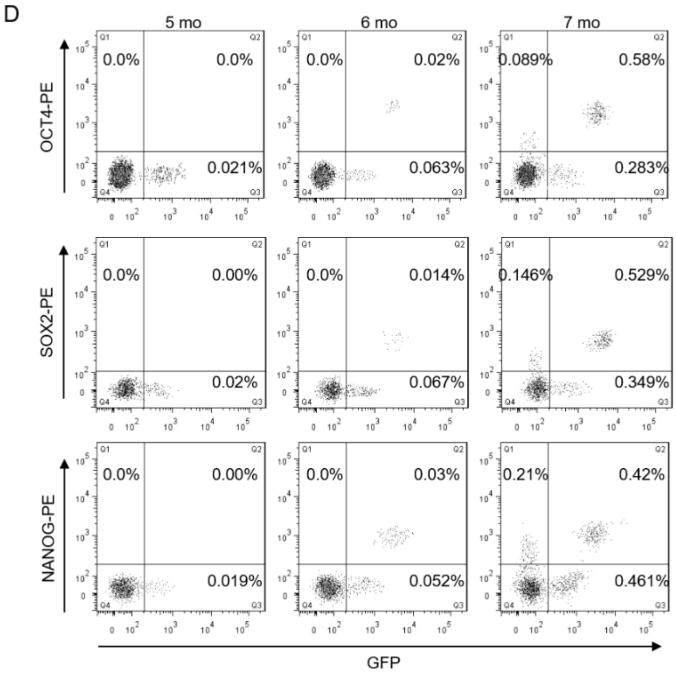

Figure S3. Related to Figure 2.

(A) Clustering of PCR array samples and heatmap displaying the fold changes of 84 CSCs-related genes between GFP$^+$ and GFP$^-$ hepatocytes. “5/6/7 mo-GFP+” indicates GFP-labelled POU2F2$^+$ mouse hepatocytes which were isolated at month 5 or 6 or 7 post-DEN. “5 mo-xenograft-GFP+” indicates GFP$^+$ hepatocytes in the 5th month-related xenograft.

(B) In accordance with 2-fold change between GFP$^+$ and GFP$^-$ hepatocytes at months 5, 6 and

7 post-DEN, the up-regulated (up arrow) and down-regulated (down arrow) genes were identified in the three timepoints. Overlapping areas represent the common up- or down-regulated genes shared by two or three timepoints.

(C) The fifteen most enriched biological processes represented by 39 genes that are significantly upregulated in GFP^+ hepatocyte relative to GFP^- cells at month 7 post-DEN.

(D) Representative images of flow cytometry. Related to Figure 2C.

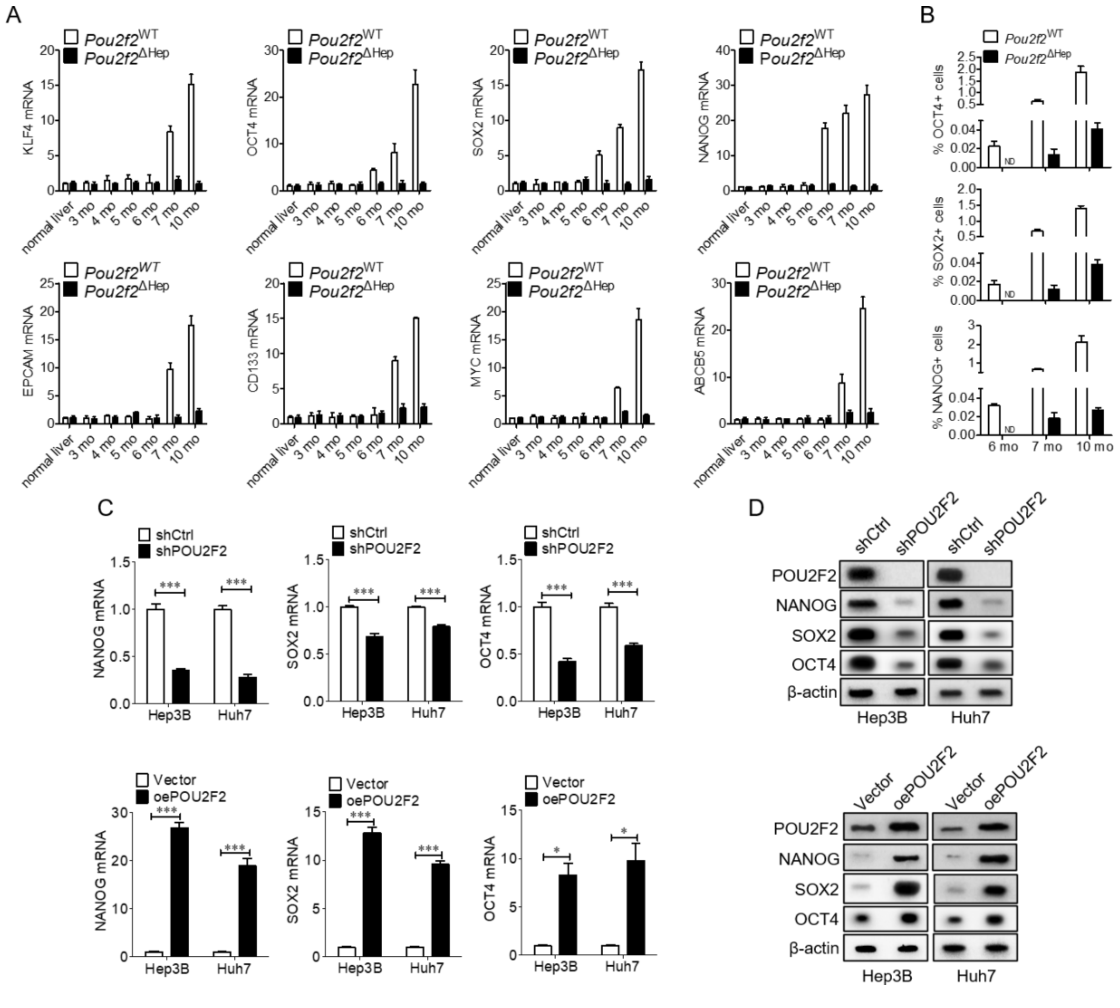

Figure S4. POU2F2 positively regulates the expression of CSCs-related genes. Related to Figure 3.

(A) Detection of 8 CSCs-related markers in the livers of *Pou2f2*$^{\Delta Hep}$ mice and control littermates (*Pou2f2*^{WT}) at months 0 (normal liver), 3, 4, 5, 6, 7 and 10 post-DEN by real-time PCR. The data are mean ± SEM, n=3.

(B) Detection $OCT4^+$ or $SOX2^+$ or $NANOG^+$ hepatocytes in *Pou2f2*$^{\Delta Hep}$ and *Pou2f2*^{WT} mice at

months 6, 7 and 10 post-DEN by flow cytometry. The data are mean ± SEM, n=3.

(C, D) In stable Hep3B and Huh7 cells expressing *POU2F2* shRNA (sh*POU2F2*) or control shRNA (shCtrl) or in stable Hep3B and Huh7 cells expressing human POU2F2 (oePOU2F2) or control vector (vector), the expressions of OCT4, SOX2 and NANOG were detected by real-time PCR (C) and immunoblot (D) assays. The data are mean ± SEM, n=3. Two-tailed *t*-test, ∗ $P<0.05$, ∗∗∗ $P<0.001$.

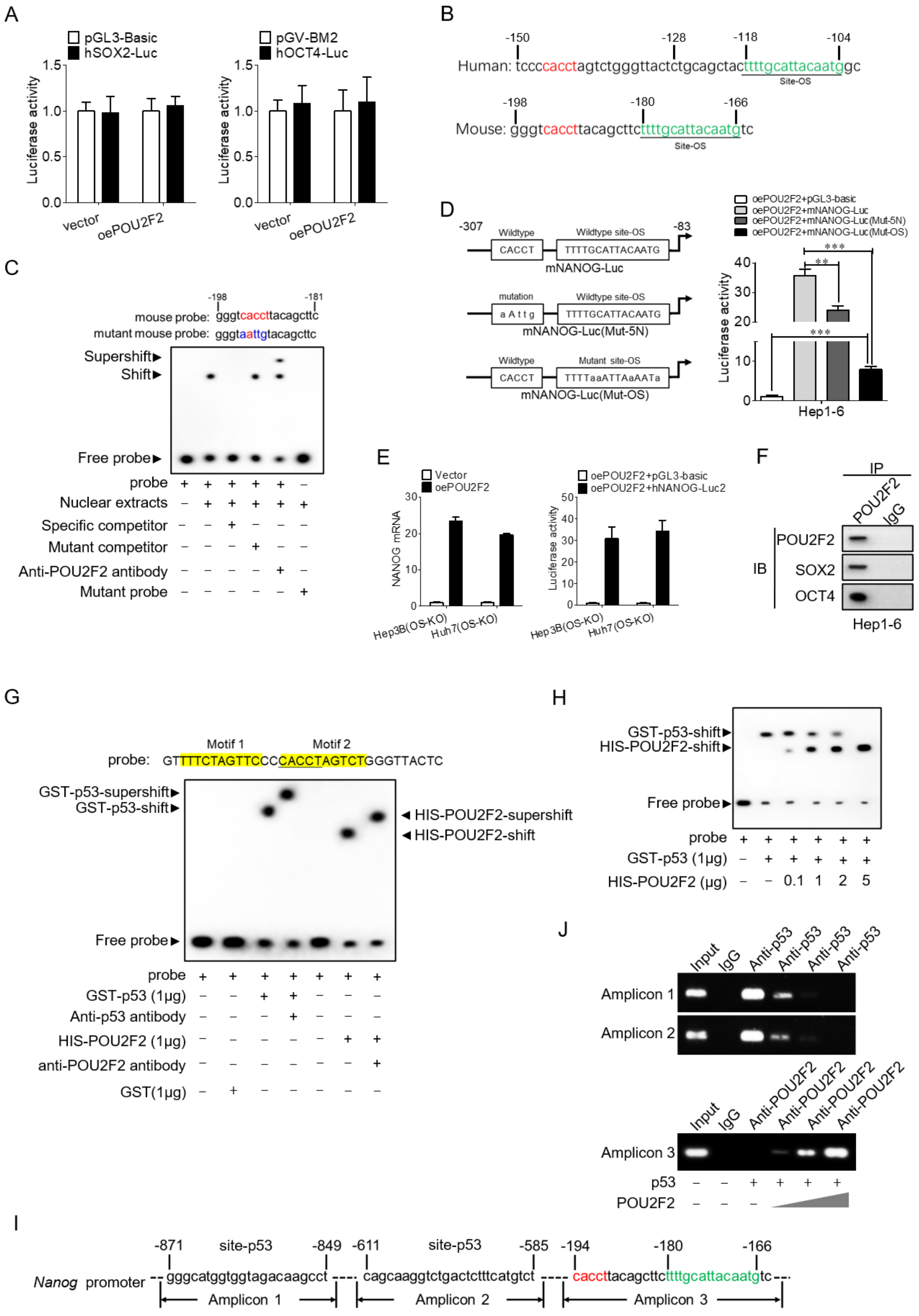

Figure S5. Related to Figure 3.

(A) Huh7 cells were transfected with human SOX2 (hSOX2-Luc, left panel) or OCT4 (hOCT4-Luc, right panel) promoter luciferase reporter illustrated together with

POU2F2-expressing plasmid (oePOU2F2) or plasmid vector (vector). The luciferase activity expressed by the pGL3-basic (left panel) or pGV-BM2 (right panel) control vector was arbitrarily defined as 1. The data are mean ± SEM, n=3.

(B) The up-stream sequence of site-OS on human or mouse *NANOG* promoter.

(C) Detection of the interaction between POU2F2 and the -198~-181 sequence of mouse *Nanog* promoter by EMSA. Nuclear extract was isolated from Hep1-6 cells. The specific competitor used was the nonlabelled probe. An anti-POU2F2 antibody was used for the super-shift assay.

(D) Hep1-6 cells were transfected with mouse *Nanog* promoter luciferase report (mNANOG-Luc) or its mutant with mutated site-OS (mNANOG-Luc(Mut-OS)) or mutated CACCT sequence (mNANOG-Luc(Mut-5N)) illustrated together with POU2F2-expressing plasmid (oePOU2F2), followed by analysis of the luciferase activities 2 days later. The luciferase activity of pGL3-basic control vector was arbitrarily defined as 1. The data are mean ± SEM, n=3. Two-tailed *t*-test, ** P<0.01, *** P<0.001.

(E) Detection of *NANOG* mRNA by real-time PCR in Hep3B/Huh7(OS-KO) cells with or without POU2F2 overexpression (oePOU2F2) (left panel). Detection of luciferase activities of hNANOG-Luc2 in Hep3B/Huh7(OS-KO) cells in response to POU2F2 overexpression (oePOU2F2) (right panel). The luciferase activity of pGL3-basic control vector was arbitrarily defined as 1. The data are mean ± SEM, n=3.

(F) Detection of the interaction between OCT4 or SOX2 and POU2F2 by CO-IP with an anti-POU2F2 antibody or control antibody (IgG) in Hep1-6 cells.

(G) The probe sequence for EMSA (upper panel). Two motif 1 and 2 (yellow marked) constructs p53-binding site. CACCT sequence, as POU2F2-binding site, is underlined. EMSA detection (lower panel). Recombinant GST-p53 or HIS-POU2F2 is used for shift assay, and anti-p53 or anti-POU2F2 antibodies is used for the super-shift assay.

(H) The concentration of GST-p53 was fixed, EMSA detected the effects of gradual increase of HIS-POU2F2 on blocking the interaction between GST-p53 and the probe of Figure S5G.

(I) Representative image of p53-binding stie (site-p53) and CACCT sequencing (red letters) and site-OS (blue letters) on mouse *Nanog* promoter. Detection of enrichments of p53 and

POU2F2 at mouse *Nanog* promoter by ChIP. Amplicon 1/2 is ChIP-detected-region for p53 binding, and Amplicon 3 is POU2F2-binding region.

(J) Hep1-6 cells were co-transfected with a plasmid encoding mouse p53 and a plasmid encoding mouse POU2F2. ChIP detected the effects of gradual increase of POU2F2 on the interaction between p53 with two site-p53 at mouse *Nanog* promoter.

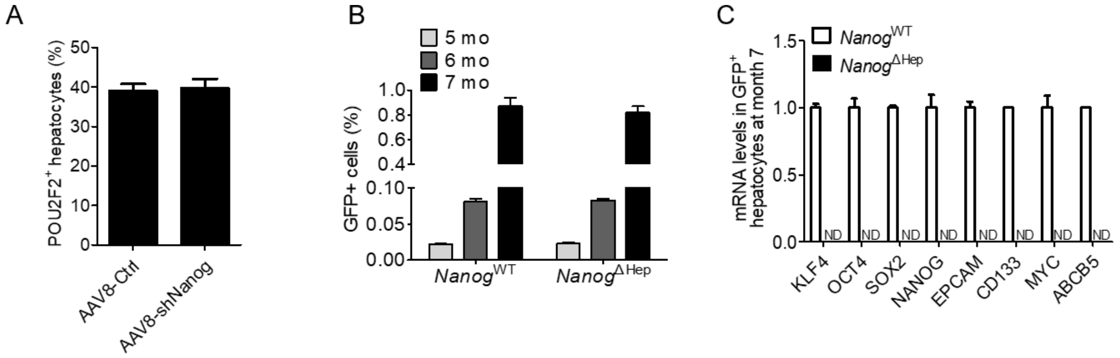

Figure S6. Related to Figure 4.

(A) DEN-challenged *Pou2f2*$^{KI\text{-}Hep}$ mice were infected with AAV8-shNanog or AAV8-shCtrl starting 1 week after DEN challenge, followed by detection of POU2F2$^+$ hepatocytes at month 10 post-TAM. The data are mean ± SEM, n=3.

(B, C) DEN-challenged *Nanog*$^{\Delta Hep}$ mice and their control littermates (*Nanog*^{WT}) were infected with Lv-P$_{mPOU2F2}$-GFP to label POU2F2$^+$ hepatocytes with GFP as in Figure 2A at months 5, 6, and 7 post-DEN, followed by detection of GFP$^+$ hepatocytes (B) or CSCs-related markers expression (C) by flow cytometry or real time PCR. The data are mean ± SEM, n=3. ND: no detection.

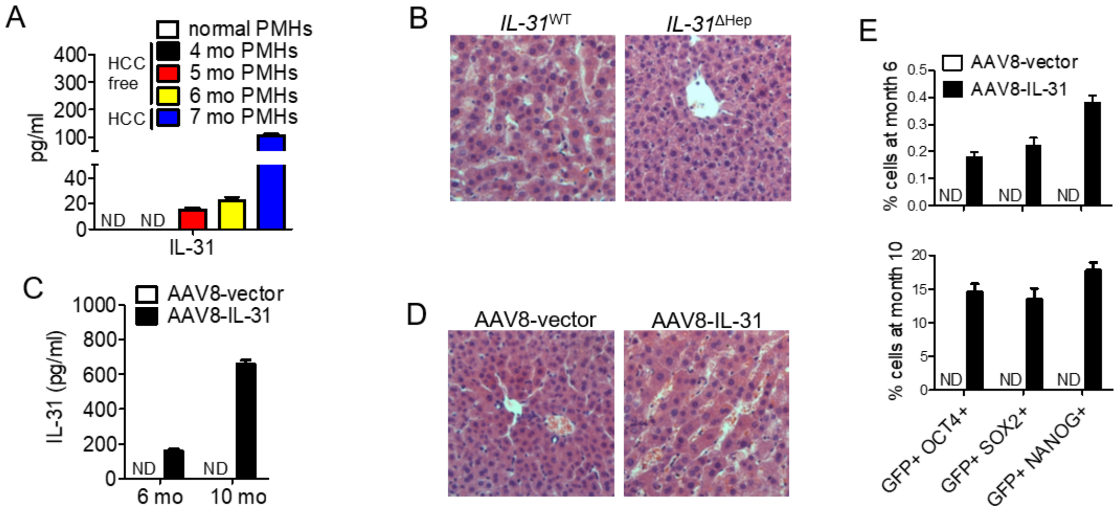

Figure S7. Related to Figure 5.

(A) PMHs were isolated from C57BL/6 mice livers at months 4, 5, 6, and 7 and then were cultured for 3 days in vitro, followed by detection of supernatant IL-31 by ELISA. PMHs isolated from the liver of ten-month-old male C57BL/6 mice that grew under normal condition were used as normal hepatocyte. DEN induced HCC development at month 7, but not months 4, 5, and 6. The data are mean ± SEM, n=3. ND: no detection.

(B) Representative H&E staining images (200×) of liver section of *IL-31*$^{\Delta Hep}$ mice and HCC section of *IL-31*^{WT} mice at month 10 post-DEN.

(C) *IL-31*$^{\Delta Hep}$ mice were infected with AAV8-IL-31 or AAV8-vector as in Figure 5G, followed by detection of IL-31 in liver homogenate by ELISA at months 6 and 10 post-DEN. The data are mean ± SEM, n=3. ND: no detection.

(D) Representative H&E staining images (200×) of HCC section of *IL-31*$^{\Delta Hep}$ mice infected with AAV8-IL-31 and liver section of *IL-31*$^{\Delta Hep}$ mice infected with AAV8-vector at month 10 post-DEN.

(E) POU2F2^{+} hepatocytes were labelled with GFP in AAV8-IL-31/AAV8-vector-infected *IL-31*$^{\Delta Hep}$ at months 6 and 10 post-DEN as in Figure 2A, followed by detection of GFP^{+} OCT4^{+} or GFP^{+} SOX2^{+} or GFP^{+} NANOG^{+} hepatocytes by flow cytometry. The data are mean ± SEM, n=3. ND: no detection.

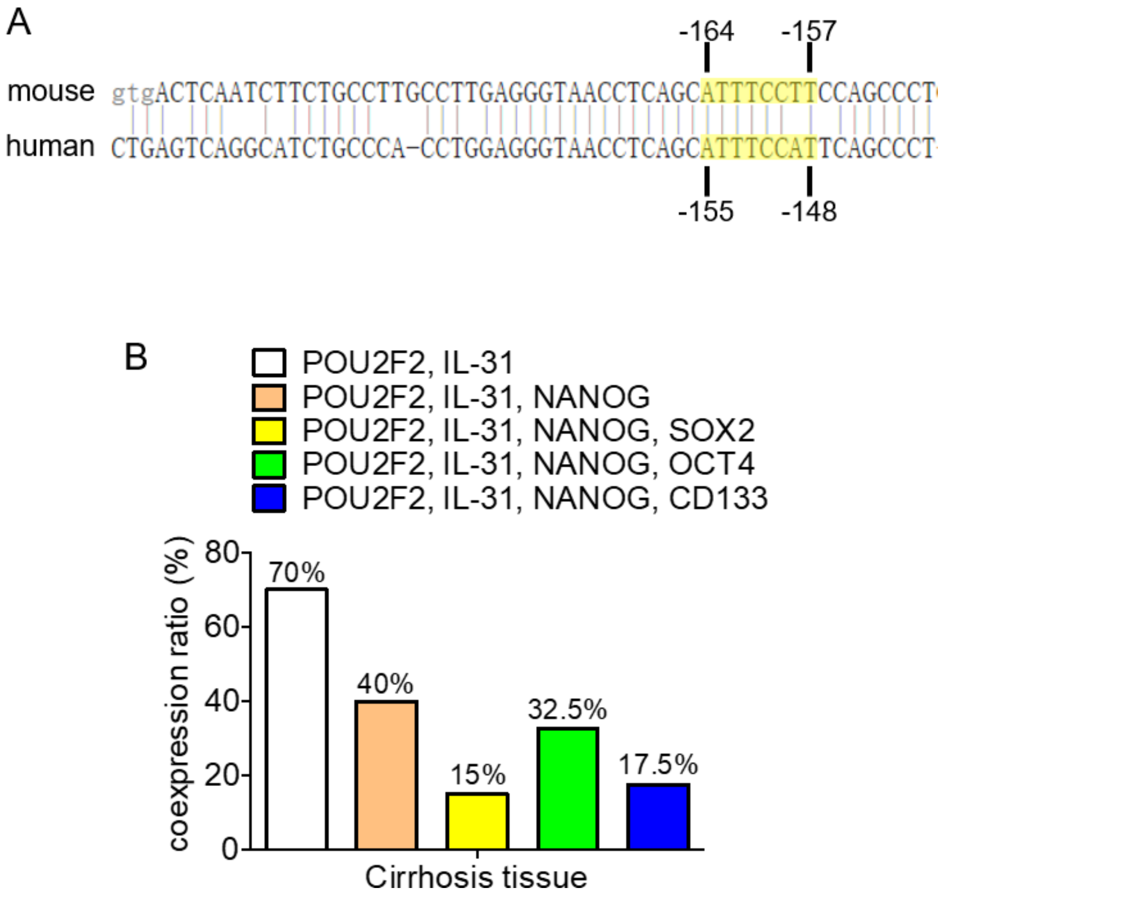

Figure S8. Related to Figure 6.

(A) Schematic representation of human and mouse *IL-31* promoter.

(B) Statistical analysis of IHC-determined the co-expression between IL-31, POU2F2, NANOG, SOX2, OCT4 and CD133. Related to Figure 6G.
